# Plant and Floret Growth at Distinct Developmental Stages During the Stem Elongation Phase in Wheat

**DOI:** 10.3389/fpls.2018.00330

**Published:** 2018-03-15

**Authors:** Zifeng Guo, Dijun Chen, Thorsten Schnurbusch

**Affiliations:** ^1^Independent HEISENBERG Research Group Plant Architecture, Leibniz Institute of Plant Genetics and Crop Plant Research, Gatersleben, Germany; ^2^Research Group Image Analysis, Leibniz Institute of Plant Genetics and Crop Plant Research, Gatersleben, Germany

**Keywords:** detillering, floret development, plant growth, stem elongation phase, wheat anther/ovary size

## Abstract

Floret development is critical for grain setting in wheat (*Triticum aestivum*), but more than 50% of grain yield potential (based on the maximum number of floret primordia) is lost during the stem elongation phase (SEP, from the terminal spikelet stage to anthesis). Dynamic plant (e.g., leaf area, plant height) and floret (e.g., anther and ovary size) growth and its connection with grain yield traits (e.g., grain number and width) are not clearly understood. In this study, for the first time, we dissected the SEP into seven stages to investigate plant (first experiment) and floret (second experiment) growth in greenhouse- and field-grown wheat. In the first experiment, the values of various plant growth trait indices at different stages were generally consistent between field and greenhouse and were independent of the environment. However, at specific stages, some traits significantly differed between the two environments. In the second experiment, phenotypic and genotypic similarity analysis revealed that grain number and size corresponded closely to ovary size at anthesis, suggesting that ovary size is strongly associated with grain number and size. Moreover, principal component analysis (PCA) showed that the top six principal components PCs explained 99.13, 98.61, 98.41, 98.35, and 97.93% of the total phenotypic variation at the green anther, yellow anther, tipping, heading, and anthesis stages, respectively. The cumulative variance explained by the first PC decreased with floret growth, with the highest value detected at the green anther stage (88.8%) and the lowest at the anthesis (50.09%). Finally, ovary size at anthesis was greater in wheat accessions with early release years than in accessions with late release years, and anther/ovary size shared closer connections with grain number/size traits at the late vs. early stages of floral development. Our findings shed light on the dynamic changes in plant and floret growth-related traits in wheat and the effects of the environment on these traits.

## Introduction

Manipulating the duration of the pre-anthesis phase is an important goal of wheat (*Triticum aestivum*) breeding for improved adaptation and yield potential (Borras-Gelonch et al., 2012; Huang et al., 2012; Romero Navarro et al., 2017). Extending the duration of the stem elongation phase (SEP, from the terminal spikelet stage to anthesis) would increase spike dry weight (DW) and the number of fertile florets at anthesis, ultimately leading to higher yields (Miralles et al., 2000; Slafer et al., 2001; Gonzalez et al., 2002), as the duration of the late SEP is associated with the number of fertile florets per spike. Also, increasing the duration of the SEP in wheat could help weaken assimilate competition and increase the number of fertile florets and grains per plant (Rahman, 1980; González et al., 2003a,b). The duration of the SEP influences assimilate allocation to the spike and to other parts of the plant, indicating that the SEP is a long, complicated phase in which many obvious phenotypic modifications can occur (e.g., floret abortion and anther/ovary swelling). Thus, it would be interesting to investigate the dynamic changes and interactions between multiple shoot growth traits (e.g., leaf, stem, spike, and tiller growth) during the SEP in wheat. Both stems and spikes grow at their fastest rates when the leaf area is declining. The association between floret death, stem length, and dry-matter growth supports the hypothesis that the death of florets is due to competition between spikes and stems for resources when the growth rate is highest (Kirby, 1988; Elhani et al., 2007; Foulkes et al., 2011; Gonzalez et al., 2011). Moreover, the rates of emergence of early-forming and later-forming leaves respond differently to photoperiod (independent, early; dependent, later). The rate of leaf primordium initiation is not significantly influenced by photoperiod, but the rate of spikelet initiation is positively associated with photoperiod, which can affect the dynamics of tillering (Miralles and Richards, 2000).

In addition to physiological analyses of the dynamic changes and interactions between all shoot growth traits, some genetic analyses have also been conducted. Leaf and spikelet initiation (LS) are strongly and positively correlated with DW at the terminal spikelet (TS) stage, but only slightly negatively correlated with DW before this stage (Borras-Gelonch et al., 2012). In addition, significant genetic correlations between LS and some tillering traits have been observed (Borras-Gelonch et al., 2012). The variation in SEP is associated with known flowering-time genes, such as *VRN* and *PPD* (Chen et al., 2009, 2010).

**Figure 1:**
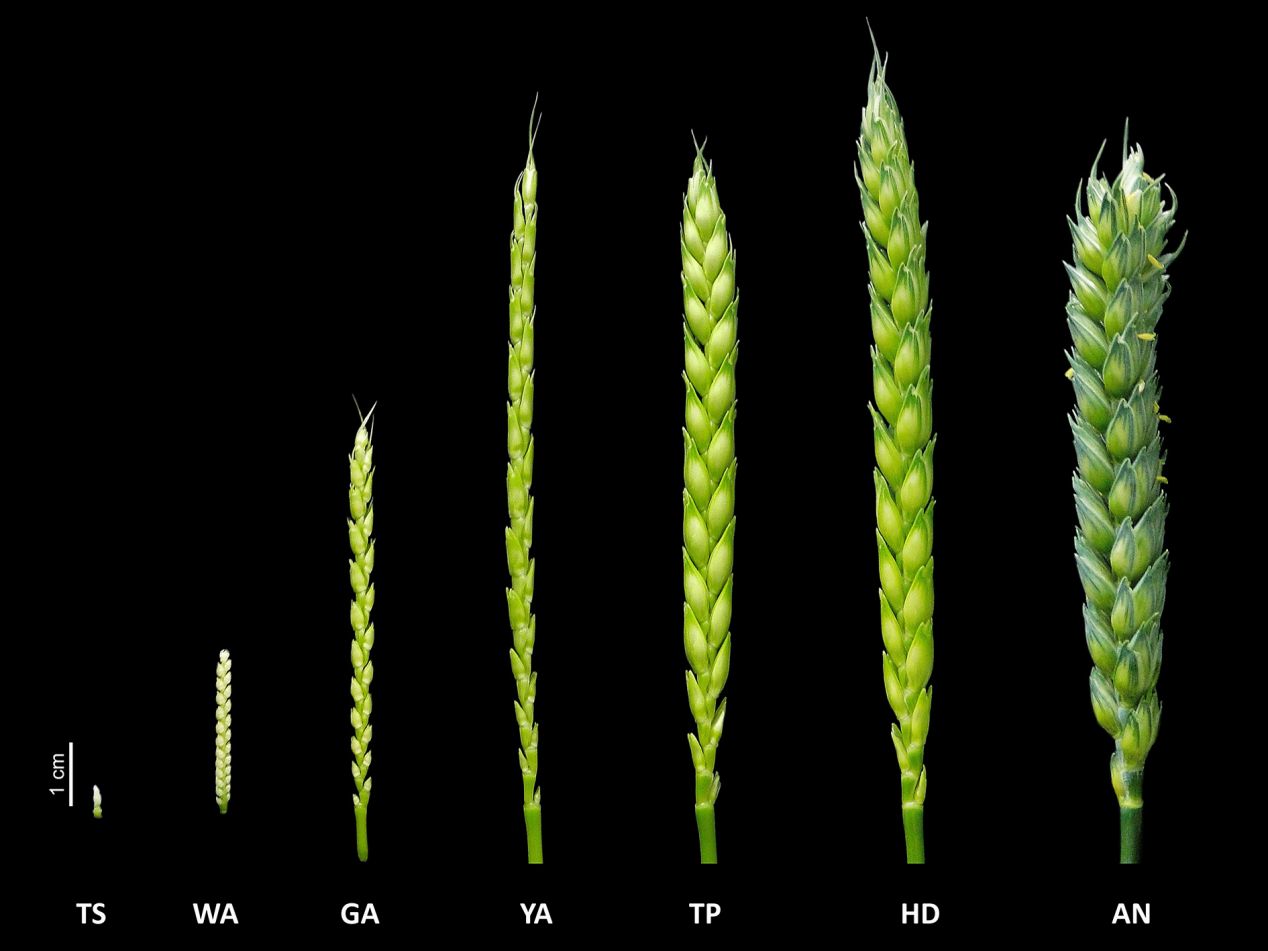
The seven floret development and abortion stages during the stem elongation phase: the terminal spikelet (TS) stage, white anther (WA) stage, green anther (GA) stage, yellow anther (YA) stage, tipping (TP) stage, heading (HD) stage, and anthesis (AN).

In this study, we investigated shoot and floret growth during the SEP in detail, as well as their correlations with grain yield traits. As the SEP is a relatively long phase, following Kirby and Appleyard (1987) and Zadoks et al. (1974), we divided it into seven stages based on the floral development and abortion process: the terminal spikelet (TS) stage, white anther (WA) stage, green anther (GA) stage, yellow anther (YA) stage, tipping (TP) stage, heading (HD) stage, and anthesis (AN) (Figure 1). The most obvious traits used to distinguish different stages were as follows: at the TS stage, no more spikelets are initiated; at the WA stage, the glumes partially enclose the florets, and the stamens and other structures cannot be detected, as floret 1 and 2 at the bottom of the spikelet are completely enclosed by their lemmas; at the GA stage, the glumes cover the entire spikelet, except for the tips of florets (Guo and Schnurbusch, 2015); at the YA stage, the glumes are fully formed and the lemmas of the first three florets are visible; at the TP stage, the first awns are visible; at the HD stage, half of an individual spike is visible; and at the AN stage, half of the spikes have yellow anthers.

Our previous analyses of the SEP (Guo and Schnurbusch, 2015; Guo et al., 2015) demonstrated that floret initiation occurs before TS until GA, floret primordia number (grain yield potential) peaks grain yield potential at the GA stage, and floret size (ovary and anther size) mainly increases from GA to AN, which is also the phase of floral abortion (Guo and Schnurbusch, 2015). In the current study, we continued to explore the SEP to obtain detailed information for investigating the dynamic growth of shoots and florets. Our analysis of the dynamic changes and interactions between shoot and floret growth based on these seven developmental stages, as well as the correlations between floret growth at the seven stages and grain yield traits, lays the foundation for further optimizing these crucial traits and could increase the efficiency of wheat breeding.

## Materials and methods

### Plant materials and growth conditions

The experiments were carried out at the Leibniz Institute of Plant Genetics and Crop Plant Research, Gatersleben, Germany (51° 49′ 23″ N, 11° 17′ 13″ E, altitude 112 m) under greenhouse and field conditions (Table S1). Thirty hexaploid winter wheat accessions (first experiment, Table S2) and 12 hexaploid spring wheat cultivars (second experiment, Table S3) were selected for phenotypic measurements. Control and tiller removal treatments were conducted in the field and greenhouse simultaneously. For detillering treatment, only the main shoot was maintained, and all other tillers were removed as soon as they became visible. In the subsequent experiment, the re-growth tillers were continuously removed two to three times per week until physiological maturity. For the control, no tillers were removed (Figure S1). Eighty plants per cultivar (40 plants for the control and 40 for tiller removal) were grown under field and greenhouse conditions.

For both the greenhouse and field experiments, seeds were sown in 96-well trays on the same date and germinated under greenhouse conditions (photoperiod, 16 h:8 h, light: dark; temperature, 20°C:16°C) for 14 days. Seedlings at the two- to three-leaf stage were transferred to 4°C and vernalized for 63 days. Vernalized seedlings were transferred to hardening conditions (photoperiod, 12 h:12 h, light:dark; temperature, 15°C) for 7 days to allow them to gradually acclimate. Finally, half of the plants were transplanted into 0.5 L pots (one plant per pot; 9 × 9 × 9 cm) under greenhouse conditions (photoperiod, 16 h:8 h, light:dark; temperature, 20°C:16°C). The plants were supplied with supplemental light (~250 mE m^−2^s^−1^ PAR, photosynthetically active radiation) from low-intensity incandescent bulbs and were irrigated when required. The remaining plants were directly planted into a field with silty loam soil (20 plants per 2 m long row with 20 cm spacing between rows). All plants were manually irrigated as required.

### Phenotypic staging and measurements

The seven floral development stages were determined according to Kirby and Appleyard (1987) and Zadoks et al. (1974): the terminal spikelet (TS) stage, when spikelet initiation was complete; the white anther (WA) stage, when the stamens of florets 1 (F1) and 2 (F2) were completely enclosed by lemmas; the green anther (GA) stage, when all but the tips of florets were covered with glumes; the yellow anther (YA) stage, when the glumes were fully formed and the lemmas of the first three florets were visible; the tipping (TP) stage (Z49), when the first awns were visible; the heading (HD) stage (Z55), when 50% of the spikes were visible; and the anthesis (AN) stage (Z65), when 50% of the spikes had yellow anthers.

To detect the TS, WA, GA, and YA stages, each genotype was examined under a stereomicroscope every 2 days (Stemi 2000-c, Carl Zeiss Micro Imaging GmbH, Gottingen, Germany). For the TP, HD, and AN stages, the day of onset was recorded as the point at which 50% of the plants reached the respective stage. Thermal time was used to identify the duration of each stage, which was calculated as the sum of the daily average temperature [(Tmax+Tmin)/2; the base temperature was assumed to be 0°C].

During each stage, three plants per cultivar were randomly selected for all phenotypic measurements. Leaf area was measured immediately after dissection of fresh main culm leaf material using an area meter (LI-3100, LI-COR Ltd., Lincoln, NE, USA). Main culm shoots and spikes were dried separately in two cellophane bags at 60°C for 3–5 days for DW measurements. Stem DW refers to the DW of one main culm shoot, including leaves without spikes. Anther and ovary size measurements were only conducted on the main culm spikes and not on the spikes of tillers. From the GA to AN stages, spikelets from the center of the main culm spike were dissected and used to obtain digital images of anthers and ovaries for three plants per stage. When the anther or ovary was aborted, the size was not measured. Anther length (anther size) and ovary width (ovary size) were also measured under a stereomicroscope using the Carl Zeiss Imaging System AxioVision Rel. 4.8.2. For most plants, only the visible structures of the anthers and ovaries in the first four florets from the base were observed. In the detillering experiment, all traits were measured on the main shoot; tiller traits were not measured.

The methods used for data analysis and statistical analysis can be found in Chen et al. (2014).

## Results

In this study, we divided plant growth into seven stages (first experiment) based on phenotypic values to obtain overall information about wheat growth in different environments. We also investigated genotypic and environmental effects on phenotypic variations in plant growth according to these seven stages. Based on the different stages that occur during the SEP, we further investigated floret growth (anther and ovary growth; five stages; second experiment) and (1) obtained information about anther and ovary size at different stages; (2) constructed a plant phenomic map and investigated phenotypic similarity based on anther and ovary growth; (3) obtained phenotypic profiles reflecting population structure according to anther and ovary growth; and (4) analyzed the associations between floret size (anther and ovary size) and grain yield traits.

### Dissecting plant growth during the SEP in wheat

**Figure 2:**
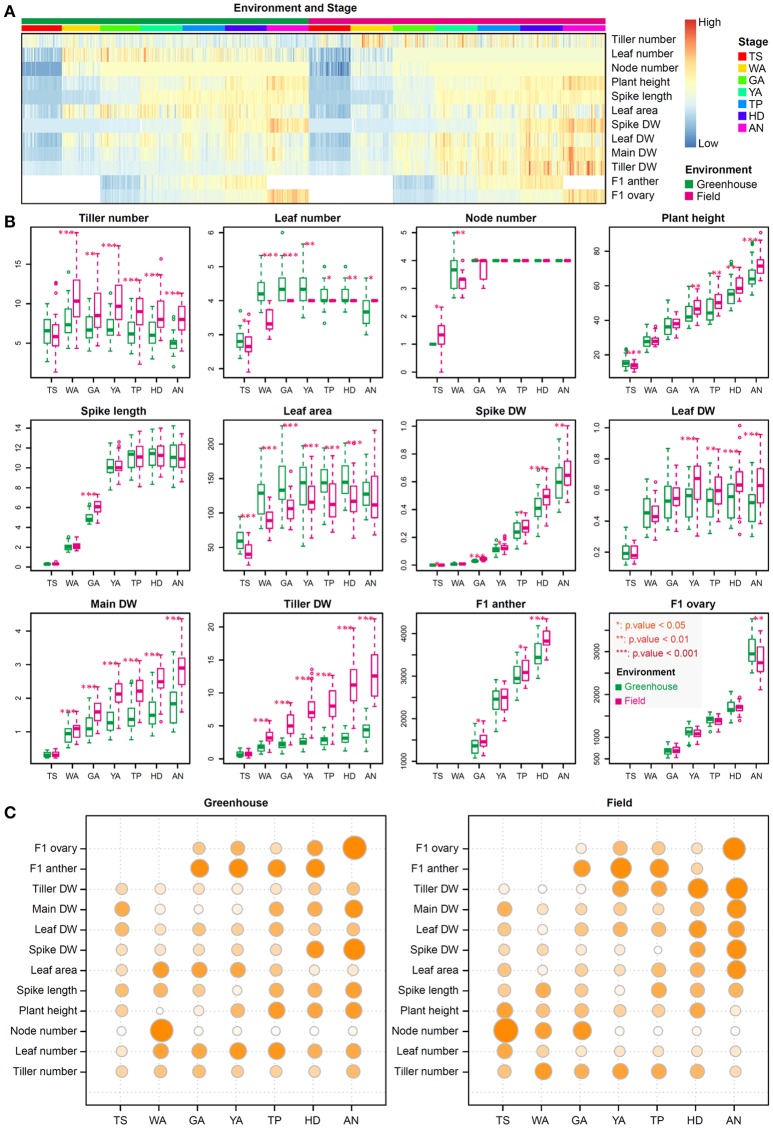
The range, trends, and relative importance of each trait at the seven stages of floral development. **(A)** Heatmap showing the relative values of the 13 measured traits. Data are scaled to zero mean and unit norm by trait (rows) across all plants (columns). Plants from different stages and/or environments (field and greenhouse) are indicated by different colors. **(B)** Boxplot showing the similarity and difference for each trait between the greenhouse and field over the seven stages investigated. The unit of measurement for plant height and spike length is centimeters, for leaf area is centimeters squared (cm^2^); for spike dry weight (DW), leaf DW, main stem DW, and tiller DW is grams (g); and for anther length and ovary width is micrometers (μm). Significant differences are indicated by asterisks (*p < 0.05; **p < 0.01; ***p < 0.001). **(C)** Bubble plots showing the relative importance of each trait at the seven developmental stages under greenhouse and field conditions. DW indicates dry weight; F1 represents the first floret from the base of the spikelet; F1 ovary and anther are the ovary and anther in the first floret from the base of the spikelet; main DW indicates dry weight of the main shoot without the spike, including the main stem and leaves. Tiller DW is the dry weight of tillers without main shoot. The high and low values in the figure are relative values for each data set.

As shown in Figure 1, we dissected the SEP into seven stages, including TS, WA, GA, YA, TP, HD, and AN, based on floral development and the abortion process. We quantified 80 plant growth traits in 30 winter wheat accessions to obtain overall information about plant growth during the SEP in both the field and greenhouse. The general range of values for most traits at the same stage was consistent between the greenhouse and field (Figure 2A). The trend in color switching was consistent for most traits, suggesting that the ranges of various plant growth traits at different stages are consistent and independent of the environment (Figure 2A).

Although the phenotypic ranges of most traits showed relatively high stability between field and greenhouse conditions, when investigating different traits at specific stages, we found that some traits significantly differed between the two environments (Figure 2B). Tiller number, leaf number, node number, leaf DW, tiller DW, and leaf area significantly differed between the greenhouse and field from WA to AN, TS to AN, TS to GA, YA to AN, WA to AN, and TS to HD, respectively. Node number did not change from GA to AN. However, leaf area was higher in the greenhouse, whereas leaf DW was higher in the field, implying that leaf thickness was higher in the field than in the greenhouse. Plant height was significantly higher at TS, YA, TP, HD, and AN in the field compared with the greenhouse, and spike DW was markedly higher under field conditions at GA, YA, TP, HD, and AN. Spike length was significantly different between greenhouse and field conditions only at the GA stage, as spike length increases rapidly at this stage and is sensitive to the environment; F1 (the first floret from the base of an individual spikelet) anthers and ovaries were significantly different between environments at AN.

The traits that could be used to distinguish between stages differed depending on the stage. In general, the distinguishing traits at different stages were consistent between the greenhouse and field, suggesting phenotypic stability, but the weights of their roles differed slightly, pointing to phenotypic plasticity (Figure 2C). Under greenhouse conditions, F1 ovary size and spike DW are two important traits for the AN stage, whereas F1 anther size plays an important role at GA, YA, TP, and HD, and node number can be used to distinguish the WA stage (Figure 2C). Under field conditions, F1 ovary size and tiller DW are two important traits for the AN stage, whereas F1 anther size is critical for GA, YA, and TP. Node number plays an important role at the TS stage, and tiller DW can be used to distinguish the HD stage. Finally, tiller DW has more influence in the field than in the greenhouse from YA to AN, and the influence of this trait increases from YA to AN under field conditions (Figure 2C).

### Genotypic and environmental effects on phenotypic variation in plant growth according to the seven stages

**Figure 3:**
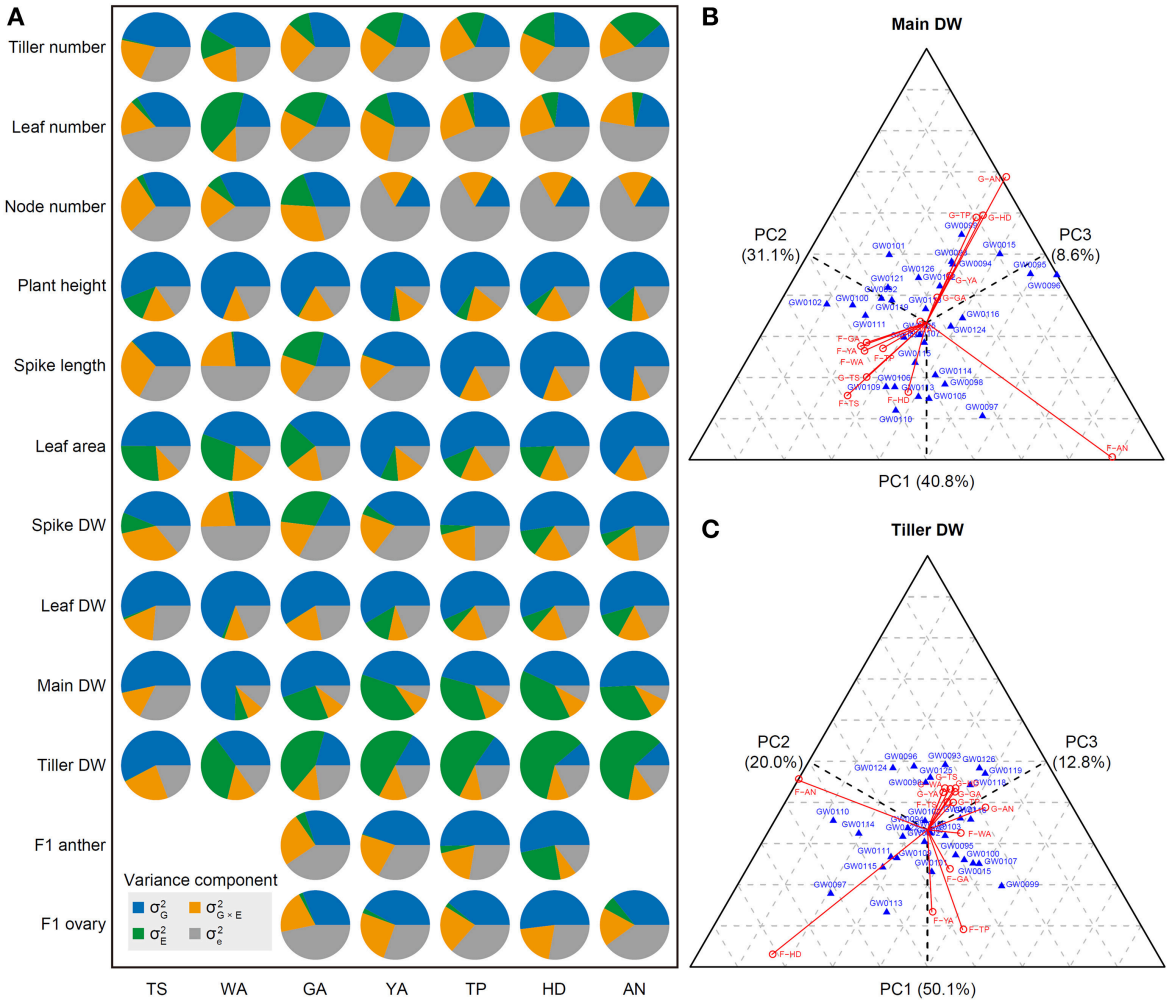
Principal component analysis of all plant growth traits according to the seven stages, and three-way principle component analysis (PCA) of main shoot DW and tiller DW. **(A)** PCA of all plant growth traits at the seven stages of the stem elongation phase. **(B)** Three-way PCA of main shoot dry weight (DW). **(C)** Three-way PCA of tiller DW. In the PCA, G and F represent greenhouse and field conditions, respectively; the length of each red line indicates the effect of a trait under greenhouse or field conditions. A strong effect for a trait at a specific stage will lead to great differences between cultivars. If cultivars are listed close to a line, these cultivars have advantages at the stage and condition indicated by the line. Main DW (in grams) indicates dry weight of the main shoot without the spike, including the main stem and leaves. Tiller DW is the dry weight of tillers without main shoot.

As shown in Figures 1, 2, most of the traits examined are significantly influenced by the environment, and different traits play different roles in distinguishing the seven stages. The influence of genotype ($\sigma_{\text{G}}^{2}$), environment ($\sigma_{\text{E}}^{2}$), genotype-environment interaction ($\sigma_{\text{G}\times E}^{2}$), and residual ($\sigma_{\text{e}}^{2}$) for different traits may explain the environmental effects and their different roles, as shown in Figures 1, 2. Therefore, we conducted variance component analysis according to the seven stages of the SEP. The results were as follows (as shown in Figure 3A): (1) the influence of genotype ($\sigma_{\text{G}}^{2}$) on tiller number showed a declining trend from TS to AN, indicating decreasing stability during this phase; (2) the genotype and environment interaction ($\sigma_{\text{E}}^{2}$) is important for determining leaf number at the WA stage, with the weight of $\sigma_{\text{G}\times E}^{2}$ decreasing from WA to AN, and genotypic effects ($\sigma_{\text{G}}^{2}$) had the strongest influence on leaf number at TS; (3) residual ($\sigma_{\text{e}}^{2}$) played an important role in determining tiller, leaf, and node number at all stages, pointing to phenotypic variation within a genotype for these three traits; (4) genotypic effect ($\sigma_{\text{G}}^{2}$) had the strongest effect (explaining >50% of variance) in PCA of plant height and leaf DW at all stages, implying that plant height is a stable trait; (5) genotypic influence ($\sigma_{\text{G}}^{2}$) played the largest role in the PCA of spike length from TP to AN; genotype-environment ($\sigma_{\text{E}}^{2}$) had almost no effect, pointing to strong stability at all three stages; (6) genotypic effects ($\sigma_{\text{G}}^{2}$) accounted for >50% of variance for all phenotypic components for leaf area from YA to AN, indicating strong stability during this phase; (7) similarly, genotypic effects ($\sigma_{\text{G}}^{2}$) accounted for the highest amount of variance in the determination of spike DW from YA to AN; (8) for main shoot DW, genotypic effects ($\sigma_{\text{G}}^{2}$) played the largest role at all stages, whereas environmental effects ($\sigma_{\text{E}}^{2}$) were quite weak; (9) environmental effects ($\sigma_{\text{E}}^{2}$) played the largest role in determining tiller DW from WA to AN, suggesting that tiller DW is highly sensitive to these effects during this phase; and (10) F1 anther and ovary size exhibited high stability at all stages examined, as revealed by the large proportion of genotypic effects ($\sigma_{\text{G}}^{2}$) and the small proportion of environmental effects ($\sigma_{\text{E}}^{2}$).

We performed three-way PCA to further dissect phenotypic variance components and to uncover their contributions to different traits at all seven stages. In three-way PCA, if the influence on a trait at a specific stage is great, this influence causes large differences between cultivars. Our results were as follows: (1) Tiller number is sensitive to the environment, with the most evident differences in tiller number between genotypes observed at stages WA and GA in the field (Figure S2). (2) The clearest differences in leaf number among genotypes were detected at YA and AN in the greenhouse, as supported by the wider range (i.e., substantial variation) in leaf number in the greenhouse vs. the field (Figure S3). (3) Genotypes could be distinguished based on node number most easily in the field at stages TS, WA, and GA (Figure S4), whereas little variation in node number was detected in the remaining stages in both the field and greenhouse. (4) The differences between genotypes based on plant height were not as obvious as those for the other traits in the field and greenhouse at all seven stages. Genotypes could be distinguished based on plant height most easily at stages TP and HD in the greenhouse and at YA and HD in the field (Figure S5), which is consistent with the finding that plant height increased a bit more rapidly in the field than in the greenhouse (Figure 2B). (5) Stages TP, HD, and AN in the greenhouse and GA, YA, and WA in the field contributed greatly to the differences in spike length detected at different stages (Figure S6). (6) For leaf area, the WA, GA and YA stages in the greenhouse and AN in the field played different roles in differentiating genotypes (Figure S7). (7) The differences in spike DW among genotypes were most obviously influenced at HD and AN in both the greenhouse and the field (Figure S8). (8) The differences in leaf DW among genotypes were much more obvious in the field environment than in the greenhouse. The greatest differences in leaf DW among genotypes were detected at the TS, YA, HD, and AN stages in the field (Figure S9). (9) The differences in main shoot DW among genotypes were most obvious at the AN stage under both greenhouse and field conditions. In addition, the differences in main shoot DW among genotypes differed at TP and HD in plants grown in the greenhouse (Figure 3B). (10) The differences in tiller DW among genotypes were much more evident in the field than in the greenhouse at all seven stages, which is in agreement with the relatively wide range of tiller DW (Figure 2B). Obvious effects on tiller DW were detected under two conditions, F-HD (field-heading) and F-AN (field-anthesis), with significant differences in tiller DW detected among the 30 wheat accessions (Figure 3C). (11) F1 anther size exhibited the most significant differences between genotypes at YA and HD in the greenhouse and at GA and TP in the field (Figure S10). (12) F1 ovary size markedly differed among genotypes at AN under both greenhouse and field conditions (Figure S11). Finally, marked differences in F1 ovary size were detected at the HD stage in the greenhouse and at the GA stage in the field and greenhouse.

### Dissecting floret (anther and ovary) growth in wheat

**Figure 4:**
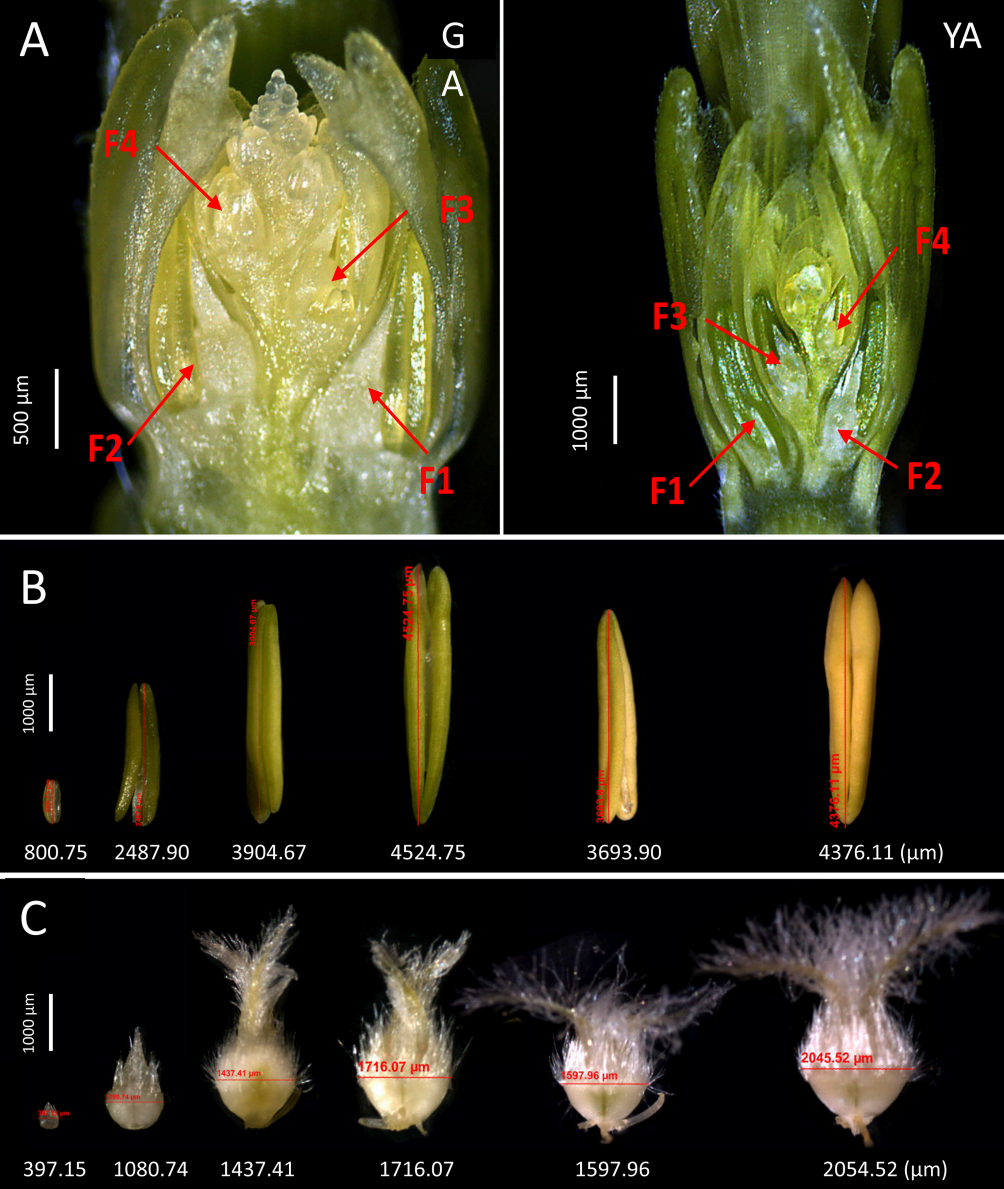
Dissection of floret growth in wheat. **(A)** Positions of the florets analyzed at green anther (GA) and yellow anther (YA) stage in this study (F1, the first floret from the bottom of an individual spikelet, F2, F3, and F4), where ovary and anther size were measured at the five floral development stages. **(B)** The six examples of measurements for anther size in this study. **(C)** The six examples of measurements for ovary size in this study.

Based on the seven stages shown in Figures 1, 2, we further investigated the five stages of anther and ovary size, as we found that anther and ovary size increase from GA (maximum number of floret primordia, grain yield potential) to AN (Guo et al., 2015). We previously found that ovary size at AN is closely associated with grain number per spike and that floral abortion occurs after GA (mainly from GA to AN) likely due to the competition for assimilates between florets within an individual spikelet (Guo et al., 2016). To further dissect the phenotypic components of anther/ovary size and to examine the roles of anther and ovary size in determining grain yield traits (including grain number per spike, grain weight per spike, individual grain area, individual grain width, individual grain length, and thousand kernel weight (TKW), we measured anther and ovary size across the four floret positions (Figures 4A–C, F1 [the first floret from the bottom of an individual spikelet], F2, F3, and F4) and five stages (GA, YA, TP, HD, and AN) under control and detillering treatment in the field and greenhouse to observe the dynamic growth of florets and the effects of detillering on floret growth.

**Figure 5:**
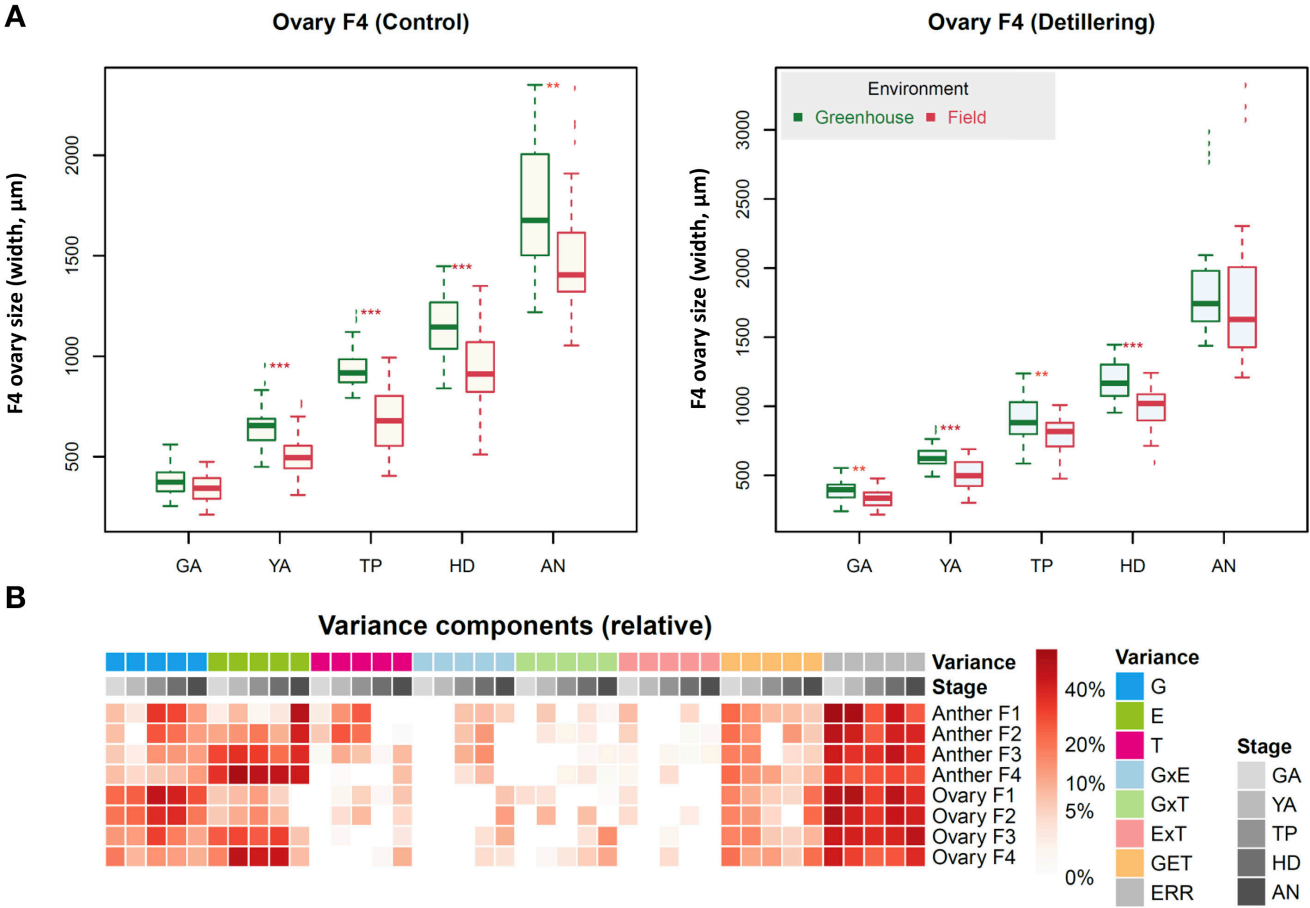
Features of anther and ovary growth across the five stages. **(A)** The effect of field and greenhouse conditions on F4 anthers and ovary size (μm) under control and detillering treatment. Significant differences are indicated by asterisks (**p < 0.01; ***p < 0.001). **(B)** Variance component analysis of F1, F2, F3, and F4 anthers and ovary size at the green anther (GA) stage, yellow anther (YA) stage, tipping (TP) stage, heading (HD) stage, and anthesis (AN). The stages, variance components, and weight of variance components are indicated by different colors.

As shown in Figure 5A, favorable (greenhouse) conditions significantly increased F4 anther size at all five stages (GA, YA, TP, HD, and AN) under both control and detillering treatment. F4 ovary size at all five stages was also significantly higher under greenhouse conditions vs. the field under both control and detillering treatment, except for AN under detillering treatment. Similarly, greenhouse conditions markedly increased anther and ovary size at positions F2 and F3 at all five stages under control and detillering treatment (Figures S12–S14). However, the effects of greenhouse conditions on anther and ovary size at position F1 were not as significant as those at F2, F3, and F4 (Figures S12–S14).

As shown in Figure 5B, according to variance component analysis of anther and ovary size at F1, F2, F3, and F4, the genotypic effects (G), environmental effects (E), interaction between genotype, environment, and treatments (GET), and residual (ERR) explained a relatively high proportion of the variance compared to the other components, including interaction between genotype and environment (GE), genotype and treatments (GT), and environment and treatments (ET).

Analysis of phenotypic similarity based on anther size, ovary size, and grain size and number revealed that anther and ovary size at the same stage were close to each other, and grain number and size were close to ovary size at AN (Figure S15).

### Plant phenomic map and phenotypic similarity based on anther and ovary growth

**Figure 6:**
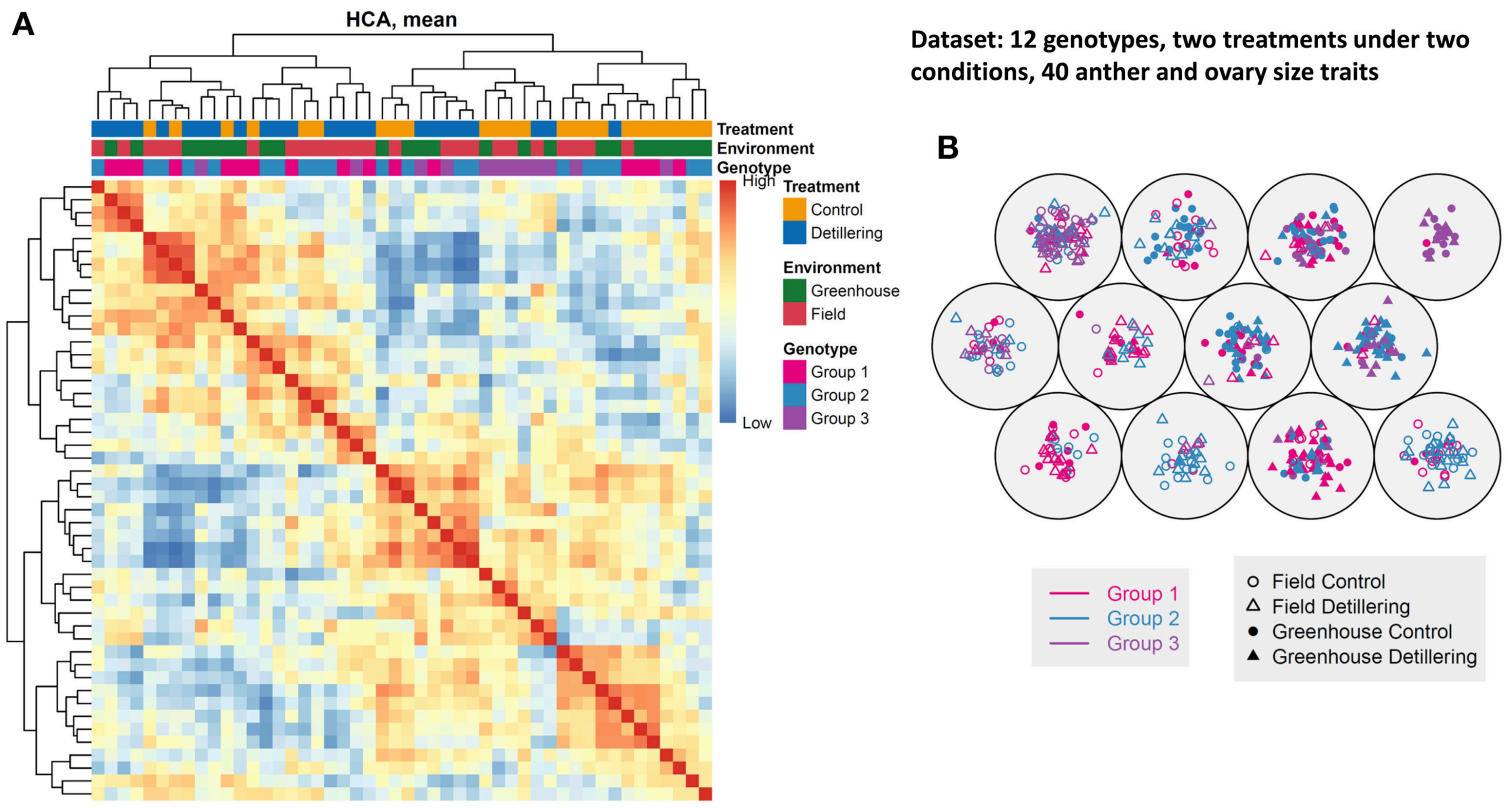
Phenotypic similarity revealed by genotypic similarity. **(A)** Hierarchical cluster analysis (HCA). Colored bars along the top of the heatmap indicate the treatments, environments, and genotype groups, as indicated. Colors on the left indicate the treatments, environments, and genotype groups, as indicated. The depth of each color indicates the relative correlation value. **(B)** A three-by-three self-organizing map (SOM). Plants under control and detillering treatment are indicated, as shown in the legend. The high and low values in the figure are relative values for each data set.

To construct a plant phenotypic map across the 12 spring wheat genotypes, we performed clustering analyses on the comprehensive phenome-wide data set across treatments (control and detillering) and environments (field and greenhouse) (Figures 6A,B). This map provides an overall view of the phenotypic similarity of plant and floret growth and facilitates further evaluation of floret growth and grain yield traits. Hierarchical cluster analysis (HCA) revealed that most traits under detillering treatment were separated from the same traits under control conditions irrespective of genotype, but the traits in the field could not be clearly differentiated from the traits in the greenhouse. To clearly visualize the phenotypic similarity among lines, as revealed by genotype similarity (the distance between genotypes in cluster analysis), we performed self-organizing map clustering analysis of the data set (Figure 6B). In the self-organizing map plot, the genotypes under detillering treatment could be distinguished from the same genotypes under control conditions, but the genotypes in the field and greenhouse could not be clearly separated. The results of HCA and self-organizing map clustering analysis support each other and are consistent with the finding that greenhouse conditions have strong effects on anther and ovary size compared to field conditions, while the differences in anther and ovary size between detillering and control treatments were not as significant as they were between the field and greenhouse.

We then constructed a neighbor-joining tree based on the 31 anther and ovary growth-related traits to investigate the phenotypic similarity of the genotypes in different release years. We constructed phenotypic similarity trees for the genotypes under greenhouse and field conditions, respectively (Figure S16). We found that genotypes with the same release years tended to cluster closely together, suggesting that anther and ovary size across different floret positions and stages are influenced by the release years of the cultivars, but the effects are not very strong. In addition, the two phenotypic similarity trees in the field and greenhouse are not very similar, but the relative relationships of some cultivars within the same groups are similar. Finally, we determined the phenotypic distance matrices of the two trees and found that they are positively associated (Pearson's coefficient *r* = 0.407, *P* < 2e-04, Mantel test; Figure S17).

### Phenotypic profile reflects population structure according to anther and ovary growth

**Figure 7:**
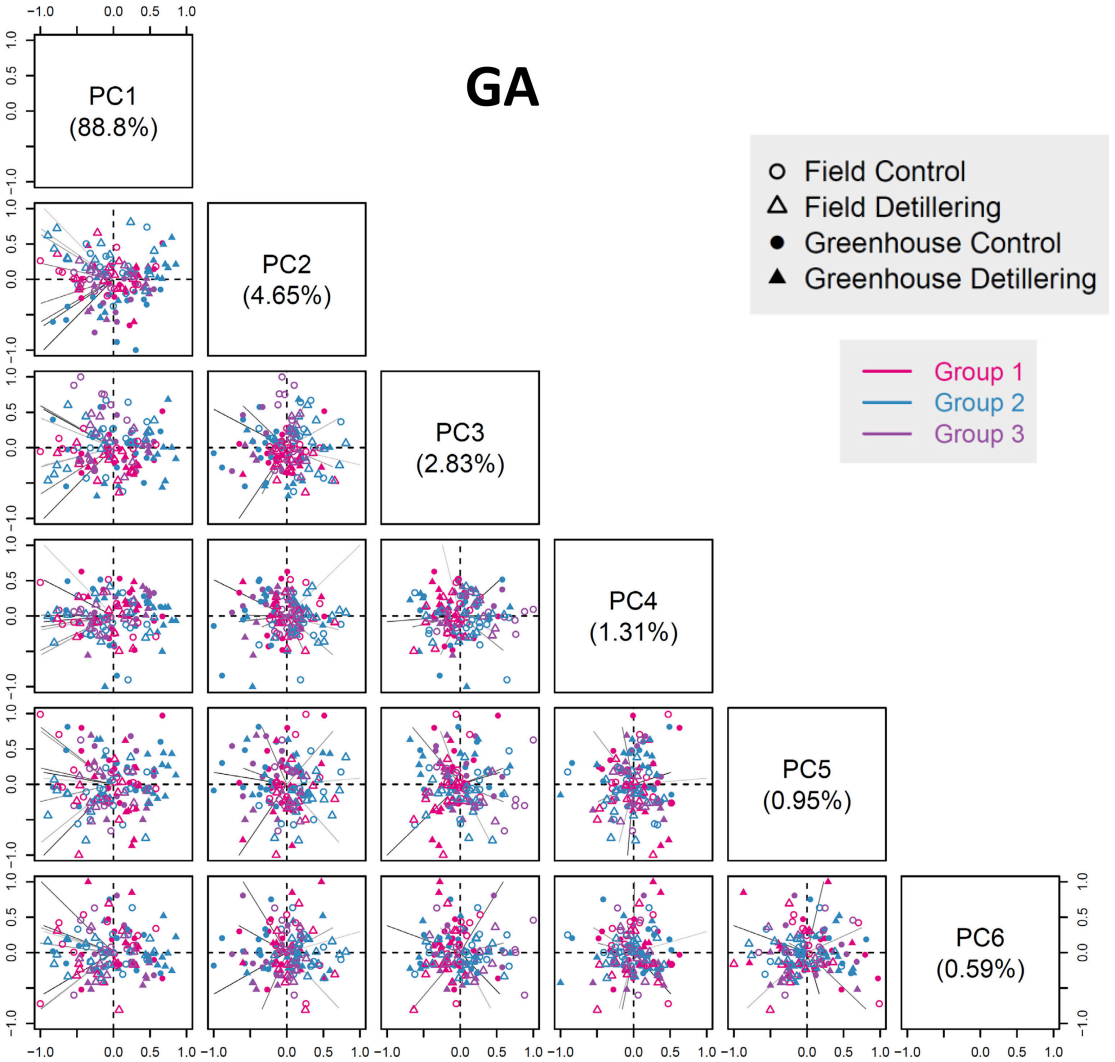
Phenotypic profile revealing population structures according to growth stage. Scatter plots showing the PCA results at the GA stage (the phenotypic variance explained by the top six PCs). The first PC explains 88.8% of the total phenotypic variance. The component scores (shown in points) are indicated by colors and shapes according to the genotype groups, treatments, and environments (see legend in the box). The length of each line (representing a component loading vector for each variable; traits are colored according to category) is proportional to the contribution of each variable.

We performed principal component analysis (PCA) to capture phenotypic variation in the entire population and to extract specific phenotypic traits regarding the discrimination of groups (Figure 7, Figures S18–S22). The top six principal components (PCs) explain 99.13, 98.61, 98.41, 98.35, and 97.93% of the total phenotypic variation at stages GA, YA, TP, HD, and AN, respectively, indicating that the plants showed the most phenotypic differences at the early growth stages. Although there were no significant differences in phenotypic variation explained by the top six PCs between the five stages, the cumulative variance explained by the top six PCs decreased with floret growth. Also, the cumulative variance explained by the first PC decreased with floret growth, with the highest explained cumulative variance detected at GA (88.8%) and the lowest at AN (50.09%).

Overall, the first PC can separate field- and greenhouse-grown plants, but it cannot distinguish between plants under control vs. detillering treatment or among the three genotype groups, whereas the other PCs cannot separate plants based on environment, treatment, or genotype. These results are in agreement with the results of clustering analysis and the finding that greenhouse conditions have strong effects on anther and ovary size compared to the field, whereas the differences in anther and ovary size between detillering and control treatments were not as significant as they were between the field and greenhouse.

### Phenotypic associations between floret size (anther and ovary size) and grain yield traits

**Figure 8:**
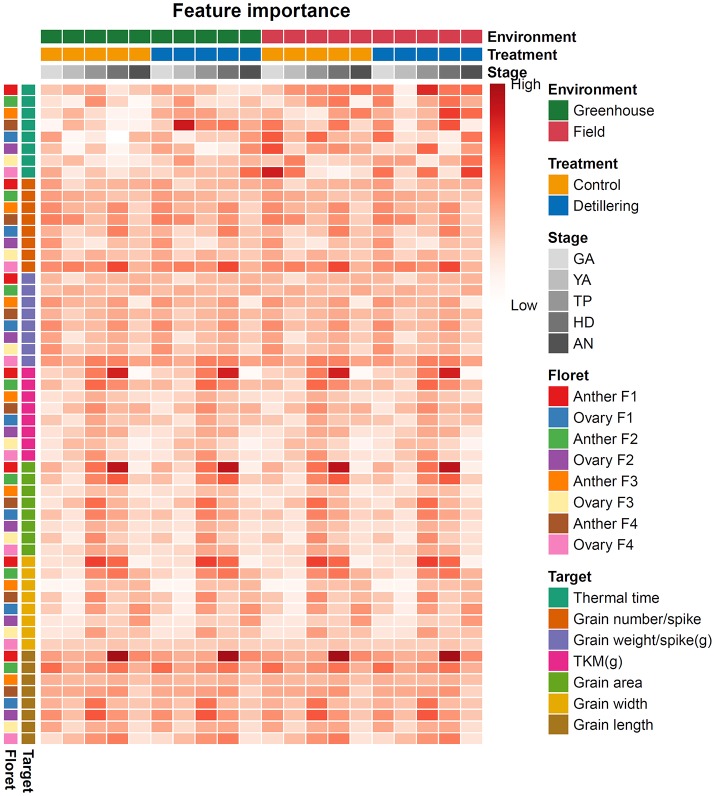
Phenotypic associations between grain yield traits and ovary (anther) size in wheat. Pearson's correlation of anther and ovary size with grain number and size traits (grain number per spike, grain weight per spike, grain area, grain width, grain length, TKW) across the five stages of flower development: the green anther (GA) stage, yellow anther (YA) stage, tipping (TP) stage, heading (HD) stage, and anthesis (AN). The relative Pearson's correlation values are indicated by the changes in color. The high and low values in the figure are relative values for each data set.

We assessed the connections between floret size (anther and ovary size) and grain yield traits (grain number per spike, grain weight per spike, grain area, grain width, grain length, TKW), as shown in Figure 8. At HD, under all four conditions (greenhouse-control, greenhouse-detillering, field-control, field-detillering), F4 ovary size was relatively closely connected with grain number per spike, suggesting that F4 ovary size at HD plays an important role in determining grain number per spike. F1 anther size was closely associated with grain size traits (TKW, individual grain area, width, length), implying that F1 anther size at HD plays a critical role in determining grain size. In addition, F1 anther size at TP was strongly correlated with individual grain width under all four conditions, indicating that F1 anther size at TP plays an important role in determining grain width. Moreover, association analysis between anther/ovary size and grain number/size showed that anther/ovary size shares relatively close connections with grain number/size traits at the late floral development stages compared with early floral development under both control and detillering treatments in both the field and greenhouse. Finally, there were no significant differences between different treatments or environments, suggesting that the connections between anther/ovary size and grain number/size are relatively stable.

### Effects of detillering and release year on anther and ovary size

As shown in Tables 1, 2, we divided the 12 spring wheat accessions into two group according to their release years (Table S3). The release years of the first six accessions range from 1931 to 1953, whereas those of the six remaining accessions range from 1959 to 1997. In the greenhouse, ovary size at anthesis at positions F1, F2, F3, and F4 in accessions with early release years was consistently larger than that of accessions with later release years. However, in the field, ovary size at anthesis in accessions with early release years was not consistently larger than that of accessions with later release years, which may be attributed to environmental influences (Tables S4, S5).

**Table 1:** Ovary size (ovary width, μm) at F1, F2, F3, and F4 under control conditions in the greenhouse.

**Control/greenhouse**	**F1 ovaries**	**F2 ovaries**	**F3 ovaries**	**F4 ovaries**
1931–1953	2908 ± 574	2622 ± 638	2053 ± 571	1548 ± 340
1959–1997	2732 ± 374	2310 ± 461	1807 ± 303	1459 ± 279
Total	2820 ± 486	2466 ± 571	1930 ± 468	1504 ± 309

**Table 2:** Ovary size (ovary width, μm) at F1, F2, F3, and F4 under detillering conditions in the greenhouse.

**Detillering/greenhouse**	**F1 ovaries**	**F2 ovaries**	**F3 ovaries**	**F4 ovaries**
1931–1953	3256 ± 679	2940 ± 702	2291 ± 722	1948 ± 668
1959–1997	2910 ± 456	2549 ± 503	1958 ± 401	1637 ± 228
Total	3083 ± 597	2744 ± 634	2125 ± 600	1793 ± 517

Finally, detillering treatment markedly increased ovary and anther size at anthesis under both greenhouse and field conditions (Tables 1, 2; Tables S4–S9). Therefore, the increased assimilate partitioning to the spike leads to improved anther and ovary size. In summary, the effects of release year and detillering treatment on anther size at anthesis were not as significant as their effects on ovary size (Tables 1, 2; Tables S4–S9).

## Discussion

The aim of this study was to dissect the stem elongation (SEP) and to investigate the phenotypic components of plant and floret growth in wheat, as the SEP is critical for determining grain yield traits, and most grain yield potential is lost during the SEP. Manipulating the duration of the SEP is an important strategy for maximizing grain yield in wheat through breeding (Gonzalez et al., 2002; González et al., 2003a,b, 2005a,b). However, most studies to date have focused on differences in yield-related traits during the entire phase of the SEP. We previously found that some SEP sub-phases are more important for improving floret fertility/grain yield than others (Guo et al., 2015). The results presented here suggest that specific stages during SEP could be targeted for manipulation, facilitating changes in plant and floret growth, improved yields and more efficient wheat breeding.

Our main purposes for measuring various plant traits were to obtain an overall understanding of plant growth, and to gauge the sensitivity and stability of the traits at different stages of growth. This information may be helpful for wheat breeders; it can help them decide at which stage to apply fertilizer for maximum floret fertility, whether to sow grains early or late to avoid stress conditions at the most important stages for grain yield, and when to manipulate various traits (e.g., leaf area) at the most important stage for maximal floret fertility/grain yield.

### Dissecting tiller and leaf growth during the SEP

Tiller and leaf growth are closely coordinated in wheat plants under favorable conditions (Bos and Neuteboom, 1998; Miralles and Richards, 2000). The low-tillering wheat genotypes first described by Atsmon and Jacobs (1977) are ideal materials for investigating tiller and leaf growth. Richards (1988) identified a single recessive gene (*tin*) located on chromosome 1A in a wheat uniculm line. Kuraparthy et al. (2007) mapped another tiller inhibition gene (*tin3*) on chromosome 3A in wheat. Physiological studies showed that *tin* lines have a greater harvest index and grain weight than the wild type, making them quite suitable for growth under terminal drought stress due to their reduced non-productive tiller number and soil water use prior to anthesis, greater biomass at anthesis, and increased water-soluble carbohydrate levels in stems (Duggan et al., 2005a,b; Mitchell et al., 2012; Hendriks et al., 2016). The flag leaves of *tin* lines are larger than those of the wild type, with longer leaf blades at higher phytomers compared to free-tillering lines, as well as reduced total leaf area (Moeller et al., 2014). The *tin* gene is positively related to root length and biomass (Hendriks et al., 2016). Precocious internode development is affected by tiller bud outgrowth in the *tin* mutant (Kebrom et al., 2012). Taken together, these findings point to the importance of tiller and leaf growth in wheat.

In the current study, we described tiller and leaf growth at a fine scale to facilitate the manipulation of the growth of these plant parts in a narrow time window to improve grain yield. We found that tiller and leaf growth were most strongly influenced by the environment (field and greenhouse) (Figure 2). PCA showed that tiller and leaf number exhibited large phenotypic variation within genotypes (high error, Figure 3A); tiller DW was sensitive to the environment (high environmental effects, Figure 3A) at all stages except TS, which is consistent with the finding that tiller DW significantly increased under field conditions at all stages except TS. Leaf area at AN and leaf DW at TS, WA, and GA were relatively stable (weak environmental effects, Figure 3A), which is supported by the observation that the environment had no significant effect on leaf area at AN or leaf DW at TS, WA, and GA.

### Dissection of spike DW, anther, and ovary size during the SEP

The importance of spike DW and both anther and ovary size in determining grain yield traits has been demonstrated in previous studies (Abbate et al., 1997; González et al., 2003a; Gonzalez et al., 2011; Fischer, 2007; Xie et al., 2015). Extending the SEP is an important way to increase spike DW at anthesis (Fischer and Stockman, 1980; González et al., 2003a, 2005a; Demotes-Mainard and Jeuffroy, 2004), which would guarantee an adequate supply of assimilate for grain filling after anthesis (Schnyder, 1993; Calderini and Reynolds, 2000; Borras et al., 2004; Ghiglione et al., 2008; Reynolds and Tuberosa, 2008). Increasing ovary size at anthesis would also improve the chances of grain setting in wheat (Guo et al., 2016). In the current study, we found that spike DW showed variable stability over the seven stages of the SEP, suggesting that the reliability of manipulating spike DW varies at different stages. For example, genotypic effects play a large role in determining spike DW at tipping, heading, and anthesis, indicating that manipulating DW would be highly reliable at these three stages. In addition, leaf area is critical for the production of photosynthetic products, which influence spike DW. Therefore, spike DW could be increased by manipulating leaf area. The high proportions of genotypic effects at the yellow anther stage and anthesis imply that it would be highly reliable to manipulate leaf area at these two stages.

Favorable conditions (greenhouse) had a significant effect on anther and ovary size, especially for anthers and ovaries at distal positions (e.g., F3, F4) within individual spikelets. This finding is in agreement with the results of variance component analysis, i.e., that environmental effects explain a large proportion of the variance in this trait (Figure 5). Moreover, this result is supported by the finding that the first PC can distinguish traits (anther and ovary size) measured in the field and greenhouse (Figure 7). Although the environment strongly affected anther and ovary size, anther and ovary size showed relatively high phenotypic similarity, since anther and ovary size at the same stages were clustered together, indicating relatively high stability. Furthermore, the moderate degree (Mantel test, *r* = 0.407, *P* = 2e-04) of correlation of phenotypic distance between genotypes under both field and greenhouse conditions and the relatively high similarity of cluster trees in the field and greenhouse indicate that both genotype and environment have relatively strong effects on anther and ovary size. These results suggest the possibility of genetically manipulating anther and ovary size and the potential risk of exposing plants to stressful environmental conditions during the floret growth phase.

Manipulating the SEP by altering the expression of known flowering-time genes (e.g., *Ppd, Vrn*) is an important way to regulate floret fertility/grain number in wheat (González et al., 2005b; Serrago et al., 2008; Ursula et al., 2017). In addition, sugar content in spikes is closely associated with floret fertility (Ghiglione et al., 2008; Dreccer et al., 2014). Although many strategies have been designed to increase grain yield in wheat, little is known about the role of anther and ovary size in determining grain yield (Guo et al., 2016). The regulation of anther and ovary growth during the pre-anthesis phase is poorly understood. It would be worth investigating the sensitivity of anther and ovary growth to different environments (e.g., heat and drought stress) in wheat.

In this study, we dissected floret growth (anther/ovary size) during the SEP to assess the key stages of floret growth to improve grain yield. Unexpectedly, we found that ovary size (ovary width in this study) at the heading stage is closely associated with grain number, while anther size (anther length in this study) at the tipping stage is closely associated with grain width. These findings indicate that ovary size at heading plays an important role in floret survival/grain setting and that anther size at the tipping stage is crucial for ovary/grain swelling. Phenotypic association analysis revealed the relatively high importance of anther and ovary size at the heading stage in determining grain number and size. We previously found that ovary size at anthesis plays an important role in determining grain number per spike (Guo et al., 2016), whereas in the current study, we found that ovary size is important during the heading stage. These different results may be attributed to environmental effects on anther and ovary size detected in this study. On the other hand, the results of this and the previous study imply that ovary size at late stages (i.e., heading and anthesis) is quite crucial for determining grain number and size.

As expected, we found that detillering clearly improved anther and ovary size, which may be attributed to the increased distribution of assimilates to grains. Unexpectedly, wheat accessions with early release years (1931–1953) had greater ovary sizes than those with later release years (1959–1997). These findings suggest that the increase in grain number that has occurred over the course of wheat breeding in the past decades may have led to a reduction in ovary size, which might further decrease grain size.

In summary, there are two important differences between the current and previous studies: (1) We studied the SEP at different developmental stages and (2) we obtained an overall view of plant and floret growth during the SEP. The analyses of phenotypes during the SEP in narrow time windows performed in this study suggest that plant and floret growth are not only controlled by phenology genes, but they are also strongly influenced by the environment. Therefore, future studies should focus on the stages of SEP in greater detail, particularly via physiological and genetic analysis.

## Author contributions

TS conceived the project; TS and ZG designed the experiments; ZG conducted phenotyping measurement in the field and glasshouse trials; DC managed the main data analysis, including figure design and generation; ZG, DC, and TS wrote the manuscript.

### Conflict of interest statement

The authors declare that the research was conducted in the absence of any commercial or financial relationships that could be construed as a potential conflict of interest. The reviewer MRT and handling Editor declared their shared affiliation.
