# Supplementary material for: Plant and Floret Growth at Distinct Developmental Stages During the Stem Elongation Phase in Wheat
Source: Front Plant Sci. 2018 Mar 15;9:330. doi: 10.3389/fpls.2018.00330 (PMC5863346; doi:10.3389/fpls.2018.00330)
Supplement: Supplementary file 1 [file Table1.DOCX]

**Table S1.** Monthly average global solar radiation and temperature during the growing season.

| Climatic data (month) | April | May | June | July | August |
| --- | --- | --- | --- | --- | --- |
| Global solar radiation (W/m^2^) | 169.2 | 191.2 | 222.8 | 208.5 | 173.7 |
| Temperature (°C) | 11.4 | 12.9 | 16.3 | 20.3 | 16.6 |
