# Supplementary material for: Plant and Floret Growth at Distinct Developmental Stages During the Stem Elongation Phase in Wheat
Source: Front Plant Sci. 2018 Mar 15;9:330. doi: 10.3389/fpls.2018.00330 (PMC5863346; doi:10.3389/fpls.2018.00330)
Supplement: Supplementary file 2 [file Table2.DOCX]

**Table S2.** Marker information for all 30 wheat cultivars.

| Cultivar number | Cultivar name | *Rht-B1* (+/-)* | *Rht-D1* (+/-)* | *Ppd-D1* (+/-)^§^ |
| --- | --- | --- | --- | --- |
| 1 | Bussard | − | − | − |
| 2 | Tukan | − | + | + |
| 3 | Tulsa | **+** | - | + |
| 4 | Türkis | − | + | + |
| 5 | Winnetou | − | − | + |
| 6 | Zentos | − | − | + |
| 7 | Zobel | − | + | + |
| 8 | Cliff | − | + | + |
| 9 | Dream | − | − | + |
| 10 | Florett | − | + | + |
| 11 | History | − | − | + |
| 12 | Lindos | − | − | + |
| 13 | Julius | − | + | + |
| 14 | Exotic | − | + | − |
| 15 | Andalou | − | + | − |
| 16 | Loch | − | + | + |
| 17 | Acienda | − | + | − |
| 18 | Aguila | − | + | − |
| 19 | Alcazar | − | + | + |
| 20 | Allister | − | + | + |
| 21 | Arack | − | + | + |
| 22 | Arobase | − | + | + |
| 23 | Astuce | − | + | + |
| 24 | Aubusson | − | + | + |
| 25 | Autan | − | + | − |
| 26 | Azimut | − | + | − |
| 27 | Azzuro | − | + | + |
| 28 | Balance | − | + | + |
| 29 | Baltimor | − | + | + |
| 30 | Bastide | − | + | + |

*(+) indicates the presence of a semi-dwarfing allele

^§^(+) indicates the presence of the photoperiod-sensitive allele at the *Ppd-D1* locus
