# Supplementary material for: Plant and Floret Growth at Distinct Developmental Stages During the Stem Elongation Phase in Wheat
Source: Front Plant Sci. 2018 Mar 15;9:330. doi: 10.3389/fpls.2018.00330 (PMC5863346; doi:10.3389/fpls.2018.00330)
Supplement: Supplementary file 3 [file Table3.DOCX]

**Table S3.** Release years for the 12 spring wheat accessions.

| Running number | Accession name | Release year |
| --- | --- | --- |
| 1 | Adlungs Alemannen | 1931 |
| 2 | NOS Nordgau | 1933 |
| 3 | Peragis Garant | 1946 |
| 4 | Heines Peko | 1947 |
| 5 | HOHENHEIMER FRANKEN II | 1951 |
| 6 | Probat | 1953 |
| 7 | Breustedts Lera | 1959 |
| 8 | ARIN | 1962 |
| 9 | Kolibri | 1966 |
| 10 | Ralle | 1980 |
| 11 | Nandu | 1988 |
| 12 | FASAN | 1997 |
