# Supplementary material for: Plant and Floret Growth at Distinct Developmental Stages During the Stem Elongation Phase in Wheat
Source: Front Plant Sci. 2018 Mar 15;9:330. doi: 10.3389/fpls.2018.00330 (PMC5863346; doi:10.3389/fpls.2018.00330)
Supplement: Supplementary file 4 [file Table4.DOCX]

**Table S4.** Ovary size (ovary width, µm) at F1, F2, F3, and F4 under control conditions in the field.

| Control/field | F1 ovaries | F2 ovaries | F3 ovaries | F4 ovaries |
| --- | --- | --- | --- | --- |
| 1931–1953 | 2896±607 | 2619±550 | 2236±447 | 1715±298 |
| 1959–1997 | 2859±455 | 2588±480 | 2116±422 | 1788±302 |
| Total | 2878±531 | 2604±510 | 2177±433 | 1749±298 |
