# Supplementary material for: Plant and Floret Growth at Distinct Developmental Stages During the Stem Elongation Phase in Wheat
Source: Front Plant Sci. 2018 Mar 15;9:330. doi: 10.3389/fpls.2018.00330 (PMC5863346; doi:10.3389/fpls.2018.00330)
Supplement: Supplementary file 5 [file Table5.DOCX]

**Table S5.** Ovary size (ovary width, µm) at F1, F2, F3, and F4 under detillering treatment in the field.

| Detillering/field | F1 ovaries | F2 ovaries | F3 ovaries | F4 ovaries |
| --- | --- | --- | --- | --- |
| 1931–1953 | 3064±623 | 2695±627 | 2311±581 | 1916±457 |
| 1959–1997 | 3100±473 | 2763±496 | 2289±464 | 1891±436 |
| Total | 3082±545 | 2729±558 | 2300±518 | 1903±440 |
