# Supplementary material for: Plant and Floret Growth at Distinct Developmental Stages During the Stem Elongation Phase in Wheat
Source: Front Plant Sci. 2018 Mar 15;9:330. doi: 10.3389/fpls.2018.00330 (PMC5863346; doi:10.3389/fpls.2018.00330)
Supplement: Supplementary file 6 [file Table6.DOCX]

**Table S6.** Anther size (anther length, µm) at F1, F2, F3, and F4 under control conditions in the greenhouse.

| Control/greenhouse | F1 anthers | F2 anthers | F3 anthers | F4 anthers |
| --- | --- | --- | --- | --- |
| 1931–1953 | 3641±400 | 3925±420 | 3685±310 | 3243±353 |
| 1959–1997 | 3550±276 | 3589±282 | 3580±381 | 3266±173 |
| Total | 3593±338 | 3757±391 | 3631±347 | 3255±271 |
