# Supplementary material for: Plant and Floret Growth at Distinct Developmental Stages During the Stem Elongation Phase in Wheat
Source: Front Plant Sci. 2018 Mar 15;9:330. doi: 10.3389/fpls.2018.00330 (PMC5863346; doi:10.3389/fpls.2018.00330)
Supplement: Supplementary file 7 [file Table7.DOCX]

**Table S7.** Anther size (anther length, µm) at F1, F2, F3, and F4 under detillering conditions in the greenhouse.

| Detillering/greenhouse | F1 anthers | F2 anthers | F3 anthers | F4 anthers |
| --- | --- | --- | --- | --- |
| 1931–1953 | 3596±320 | 3936±330 | 3931±354 | 3386±480 |
| 1959–1997 | 3683±219 | 3839±327 | 3913±375 | 3596±353 |
| Total | 3639±274 | 3887±327 | 3922±359 | 3491±429 |
