# Supplementary material for: Plant and Floret Growth at Distinct Developmental Stages During the Stem Elongation Phase in Wheat
Source: Front Plant Sci. 2018 Mar 15;9:330. doi: 10.3389/fpls.2018.00330 (PMC5863346; doi:10.3389/fpls.2018.00330)
Supplement: Supplementary file 8 [file Table8.DOCX]

**Table S8.** Anther size (anther length, µm) at F1, F2, F3, and F4 under control conditions in the field.

| Control/field | F1 anthers | F2 anthers | F3 anthers | F4 anthers |
| --- | --- | --- | --- | --- |
| 1931–1953 | 4257±347 | 4338±472 | 4262±492 | 3711±578 |
| 1959–1997 | 3934±260 | 4185±252 | 4036±294 | 3872±264 |
| Total | 4100±345 | 4263±384 | 4152±418 | 3787±459 |
