# Supplementary material for: Plant and Floret Growth at Distinct Developmental Stages During the Stem Elongation Phase in Wheat
Source: Front Plant Sci. 2018 Mar 15;9:330. doi: 10.3389/fpls.2018.00330 (PMC5863346; doi:10.3389/fpls.2018.00330)
Supplement: Supplementary file 9 [file Table9.DOCX]

**Table S9.** Anther size (anther length, µm) at F1, F2, F3, and F4 under detillering conditions in the field.

| Detillering/field | F1 anthers | F2 anthers | F3 anthers | F4 anthers |
| --- | --- | --- | --- | --- |
| 1931–1953 | 4258±301 | 4441±250 | 4412±222 | 4029±269 |
| 1959–1997 | 3921±343 | 4091±316 | 4157±403 | 3873±265 |
| Total | 4090±361 | 4266±332 | 4284±345 | 3951±275 |
