# Supplementary material for: Plant and Floret Growth at Distinct Developmental Stages During the Stem Elongation Phase in Wheat
Source: Front Plant Sci. 2018 Mar 15;9:330. doi: 10.3389/fpls.2018.00330 (PMC5863346; doi:10.3389/fpls.2018.00330)
Supplement: Supplementary file 10 [file Image1.PDF]

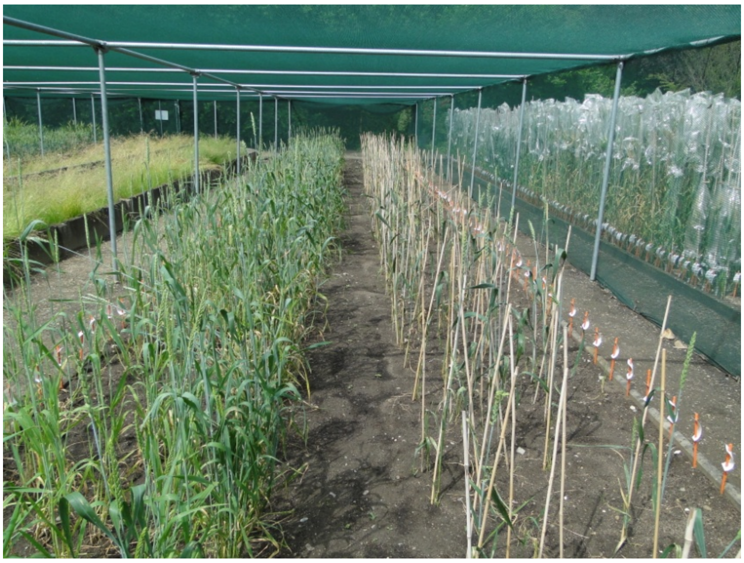

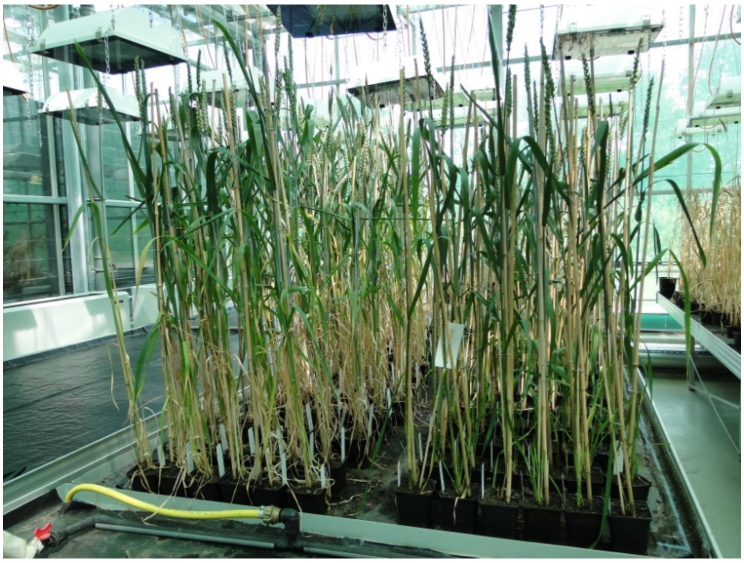

Fig. S1. Control and tiller removal experiments in field (up) and greenhouse (down).

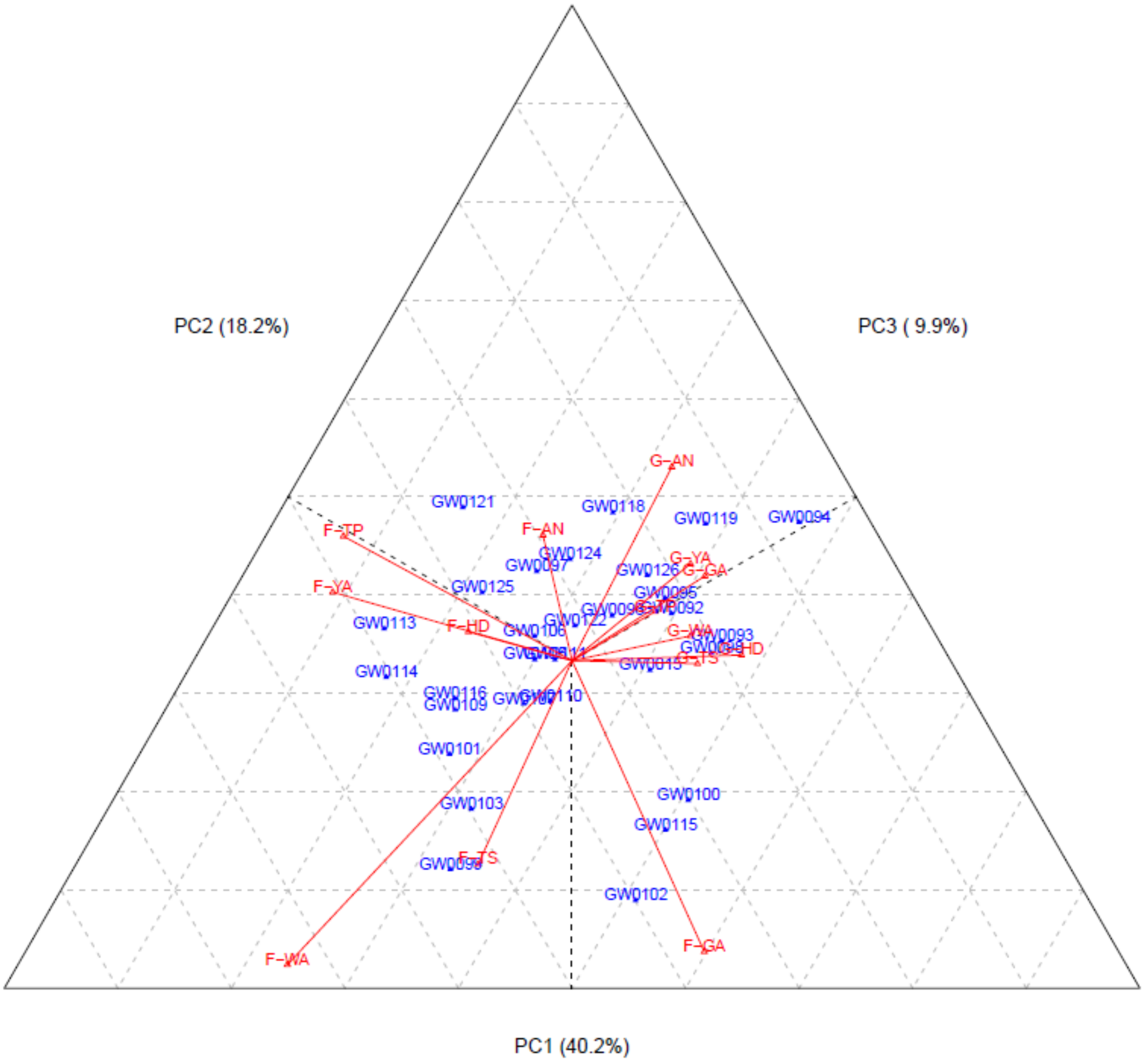

Figure S2. Three ways PCA analysis of tiller number. In PCA analysis, G and F mean greenhouse and field conditions respectively, the length of the red lines mean the influence of the different traits under greenhouse or field conditions. The influence for the traits at the specific stage is great; the influence will cause the great differences between cultivars. If cultivars are close to one line, it means these cultivars have advantages at this stage under this condition which the line shows.

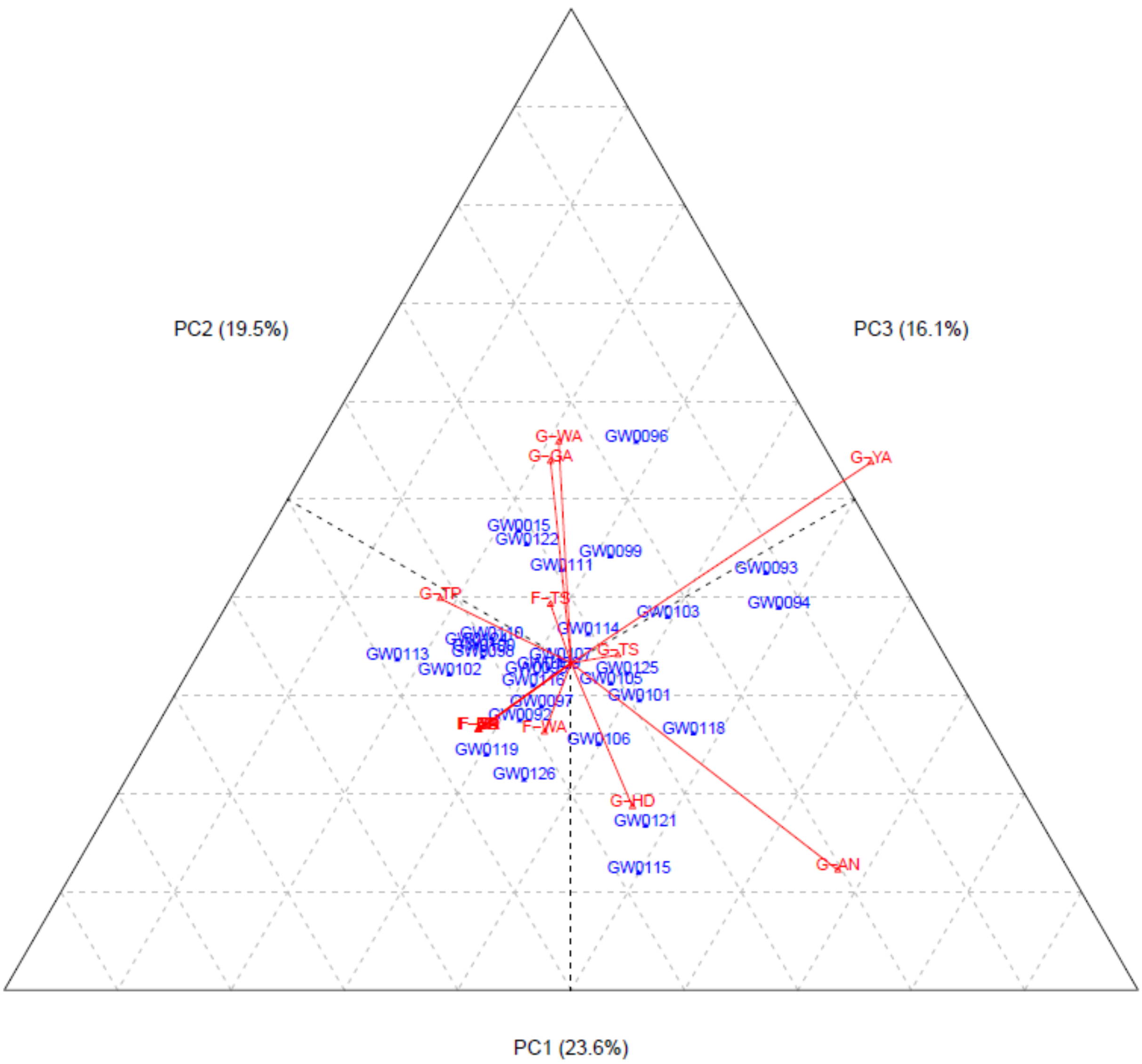

Figure S3. Three ways PCA analysis of leaf number of the main shoot. In PCA analysis, G and F mean greenhouse and field conditions respectively, the length of the red lines mean the influence of the different traits under greenhouse or field conditions. The influence for the traits at the specific stage is great; the influence will cause the great differences between cultivars. If cultivars are close to one line, it means these cultivars have advantages at this stage under this condition which the line shows.

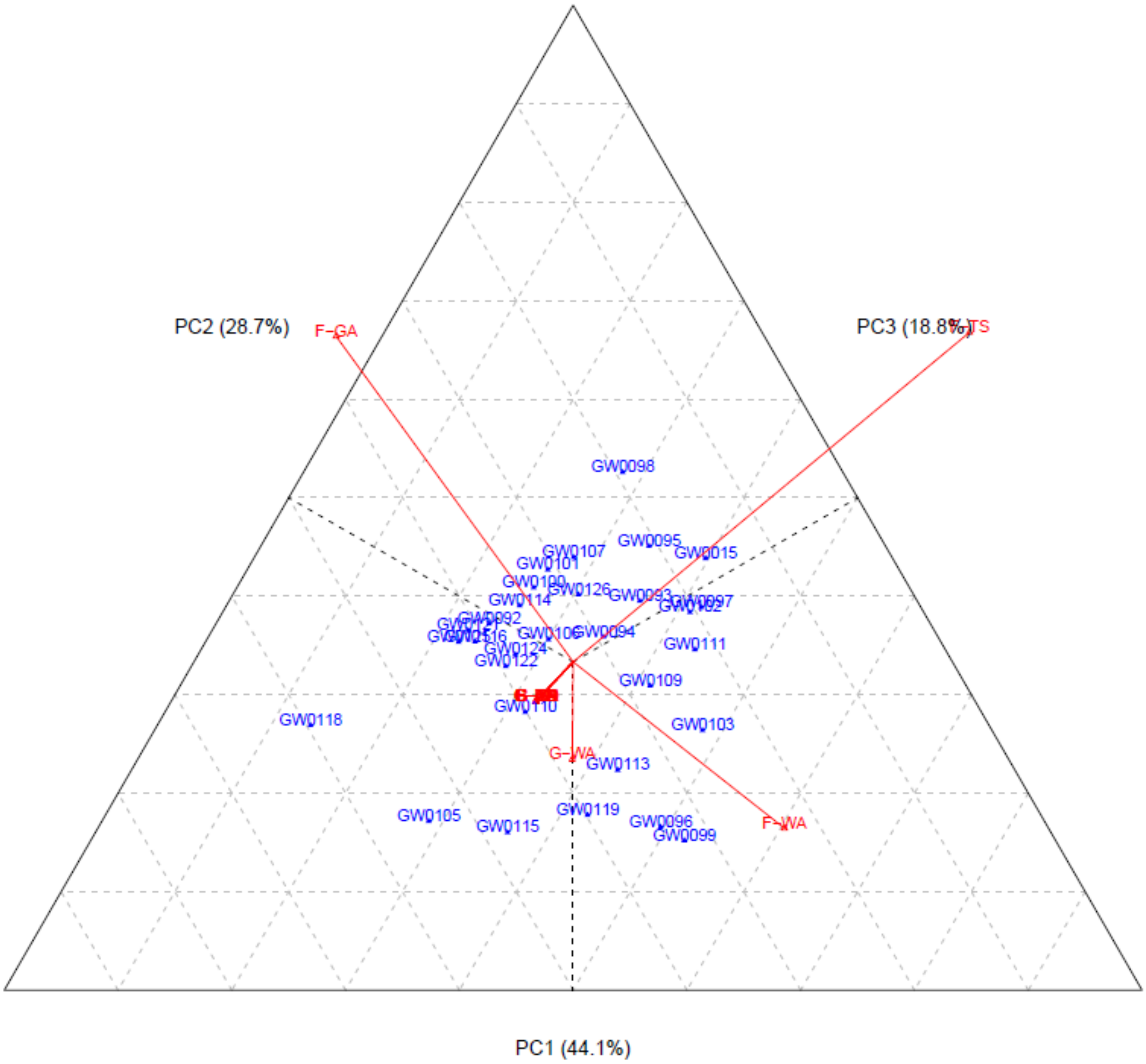

Figure S4. Three ways PCA analysis of node number of the main shoot. In PCA analysis, G and F mean greenhouse and field conditions respectively, the length of the red lines mean the influence of the different traits under greenhouse or field conditions. The influence for the traits at the specific stage is great; the influence will cause the great differences between cultivars. If cultivars are close to one line, it means these cultivars have advantages at this stage under this condition which the line shows.

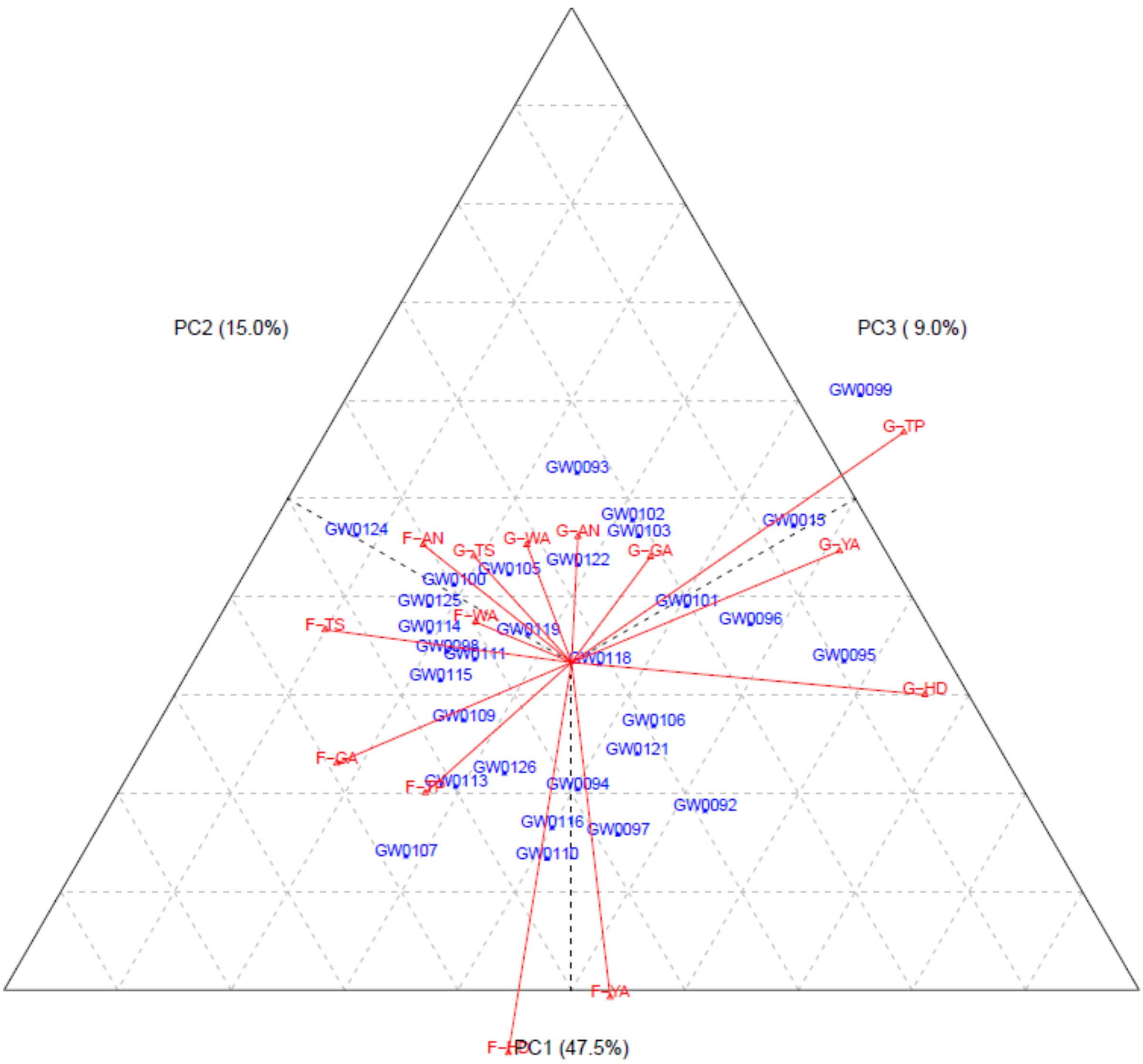

Figure S5. Three ways PCA analysis of plant height. In PCA analysis, G and F mean greenhouse and field conditions respectively, the length of the red lines mean the influence of the different traits under greenhouse or field conditions. The influence for the traits at the specific stage is great; the influence will cause the great differences between cultivars. If cultivars are close to one line, it means these cultivars have advantages at this stage under this condition which the line shows.

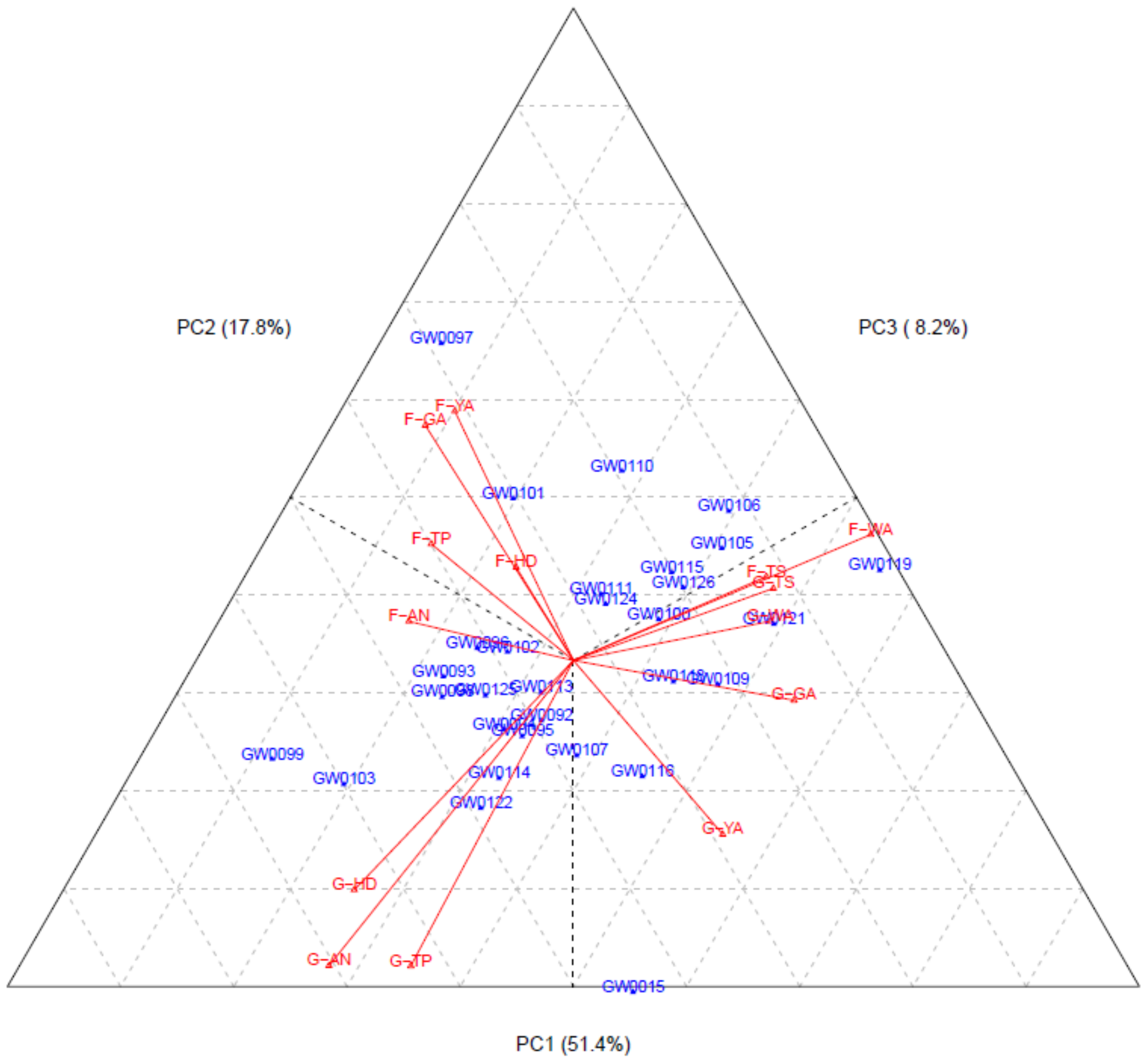

Figure S6. Three ways PCA analysis of spike length of the main shoot. In PCA analysis, G and F mean greenhouse and field conditions respectively, the length of the red lines mean the influence of the different traits under greenhouse or field conditions. The influence for the traits at the specific stage is great; the influence will cause the great differences between cultivars. If cultivars are close to one line, it means these cultivars have advantages at this stage under this condition which the line shows.

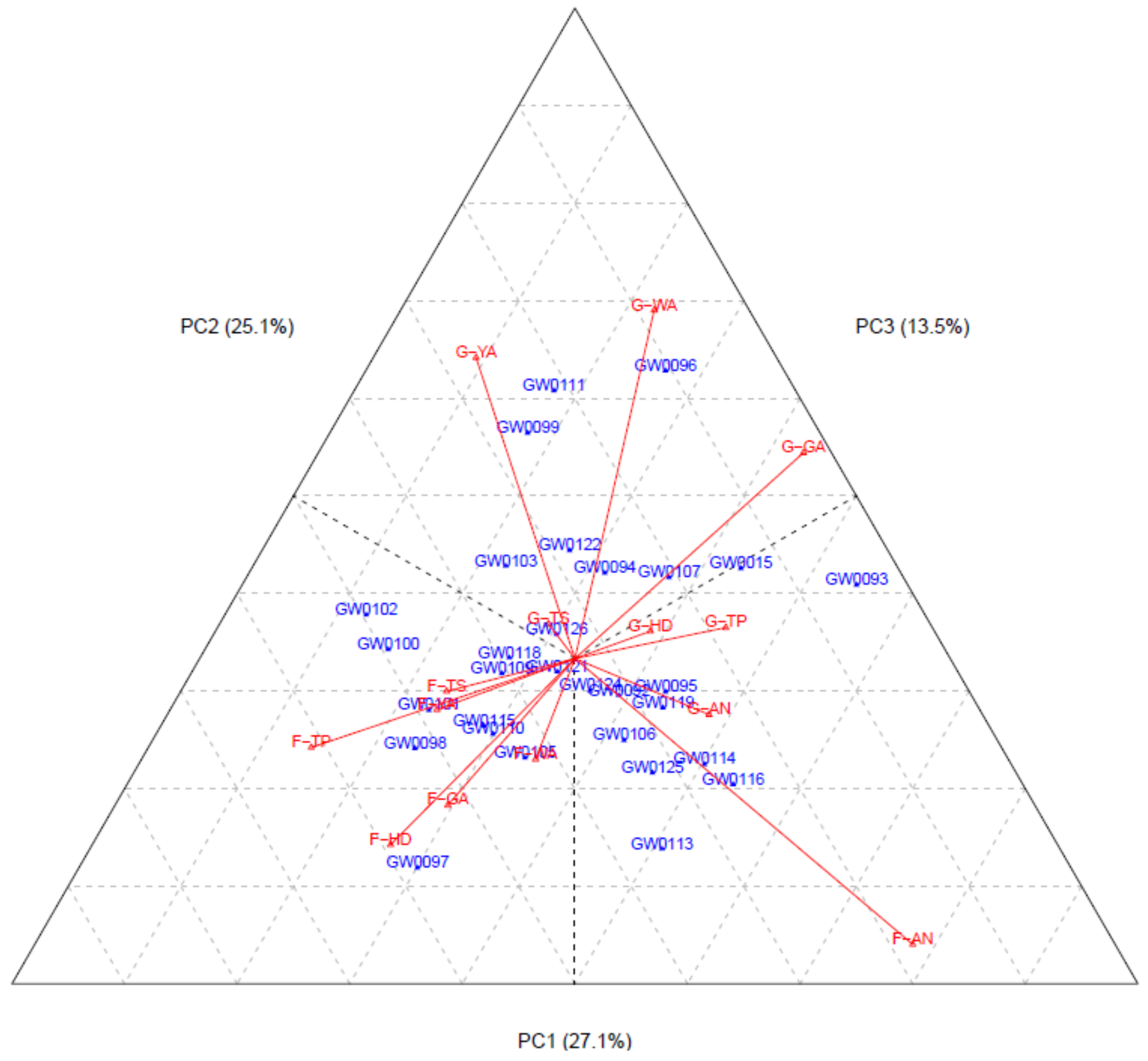

Figure S7. Three ways PCA analysis of leaf area of the main shoot. In PCA analysis, G and F mean greenhouse and field conditions respectively, the length of the red lines mean the influence of the different traits under greenhouse or field conditions. The influence for the traits at the specific stage is great; the influence will cause the great differences between cultivars. If cultivars are close to one line, it means these cultivars have advantages at this stage under this condition which the line shows.

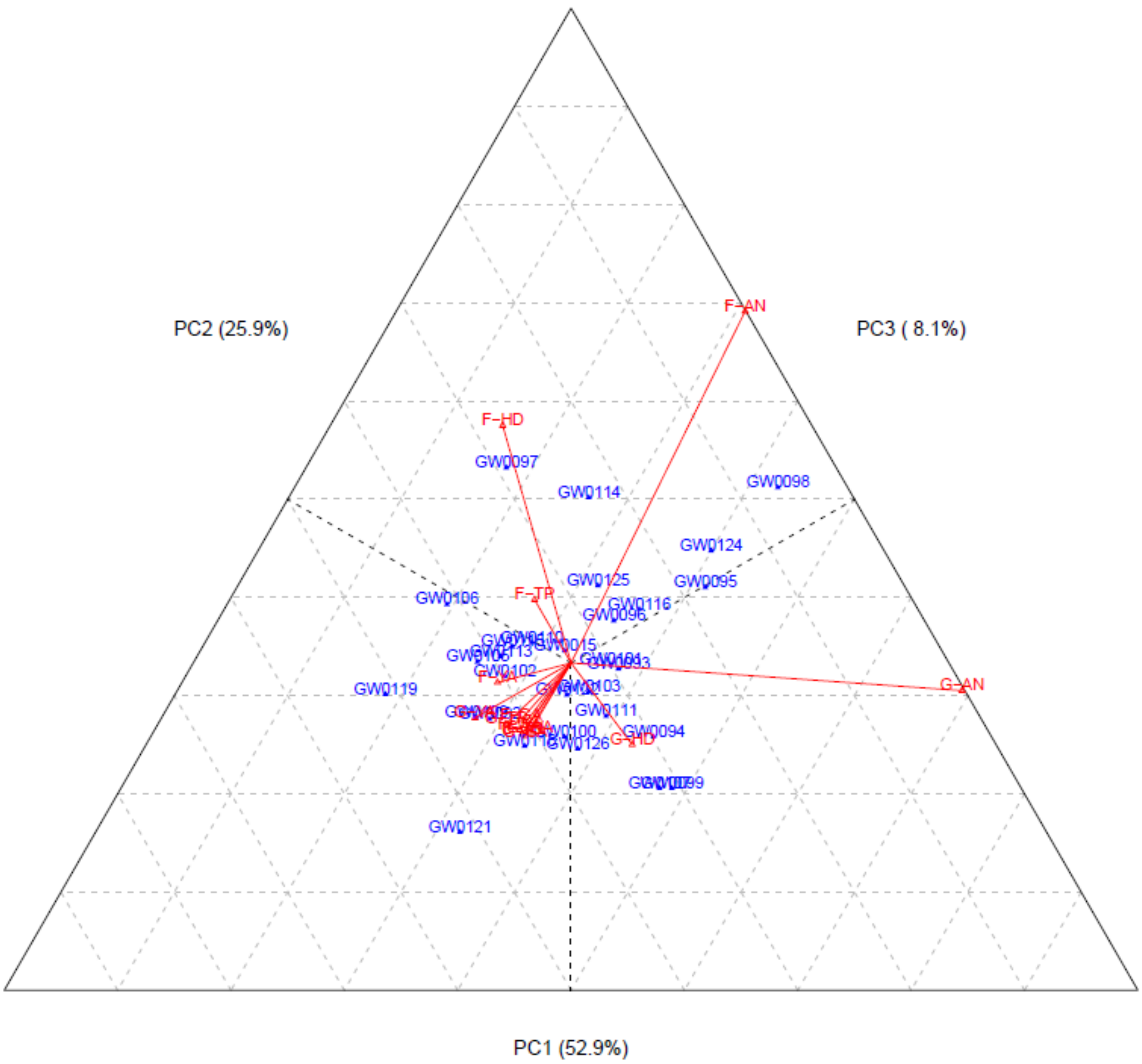

Figure S8. Three ways PCA analysis of spike dry weight (DW) of the main shoot. In PCA analysis, G and F mean greenhouse and field conditions respectively, the length of the red lines mean the influence of the different traits under greenhouse or field conditions. The influence for the traits at the specific stage is great; the influence will cause the great differences between cultivars. If cultivars are close to one line, it means these cultivars have advantages at this stage under this condition which the line shows.

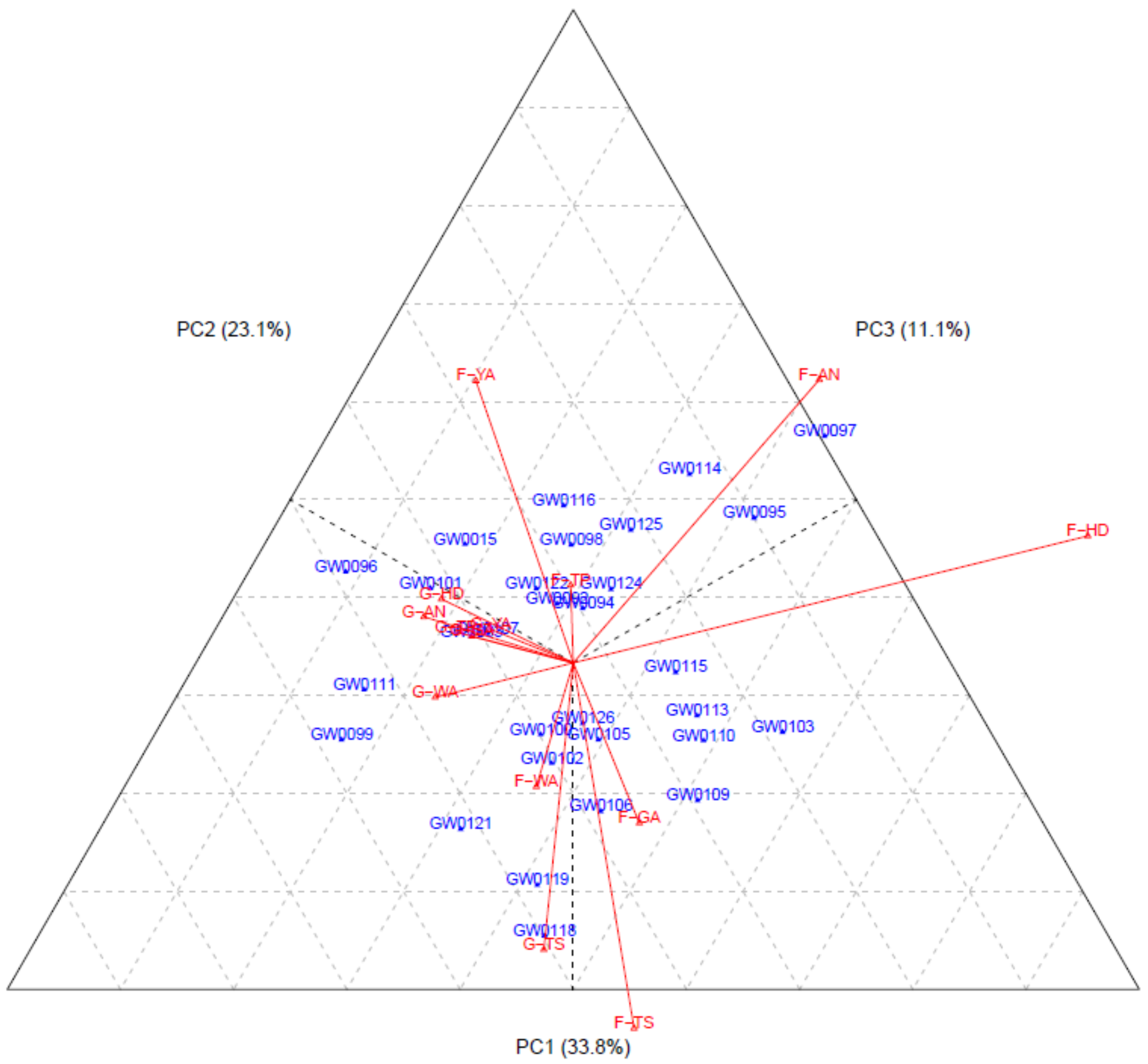

Figure S9. Three ways PCA analysis of leaf dry weight (DW) of the main shoot. In PCA analysis, G and F mean greenhouse and field conditions respectively, the length of the red lines mean the influence of the different traits under greenhouse or field conditions. The influence for the traits at the specific stage is great; the influence will cause the great differences between cultivars. If cultivars are close to one line, it means these cultivars have advantages at this stage under this condition which the line shows.

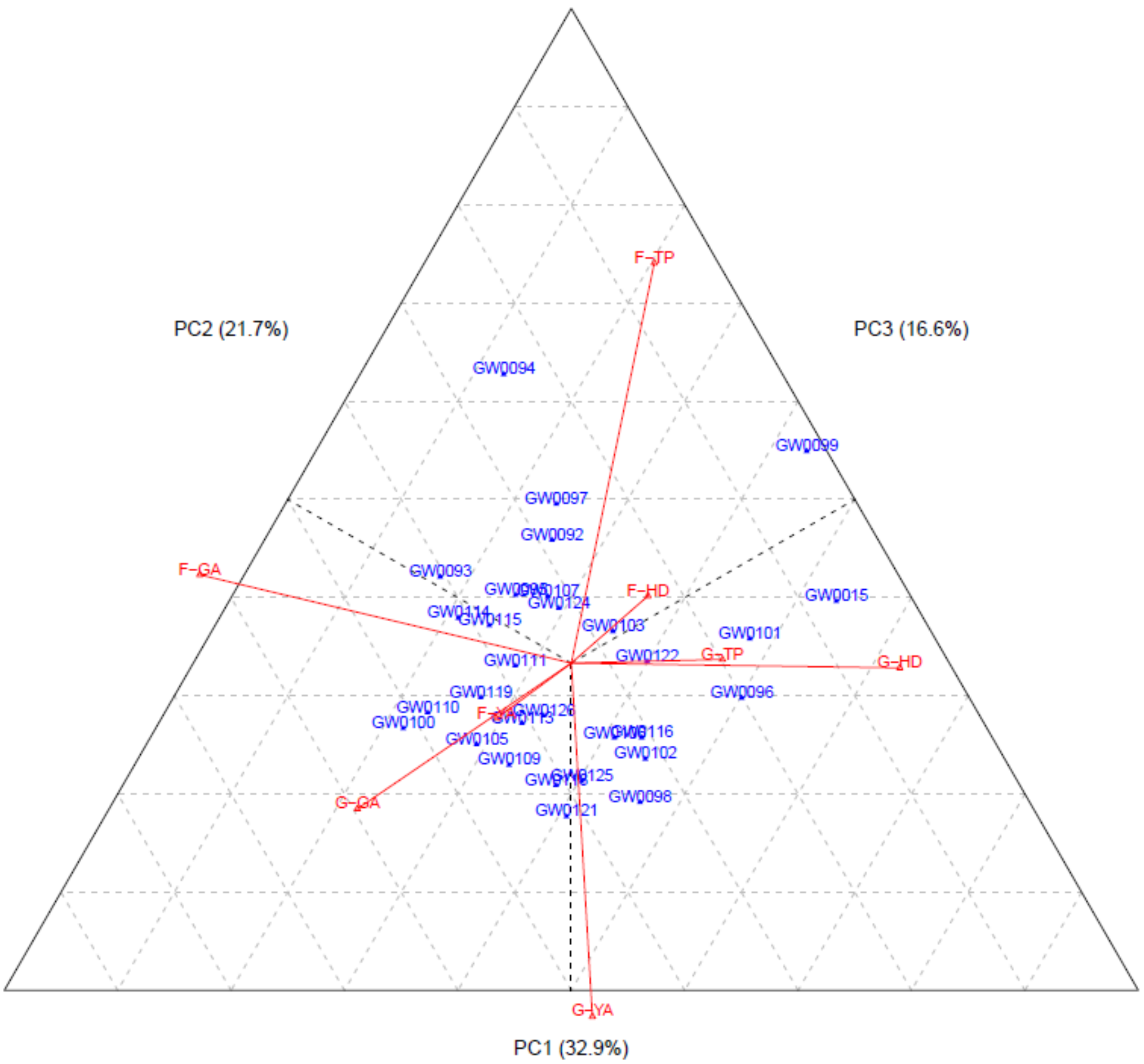

Figure S10. Three ways PCA analysis of F1 anther size. In PCA analysis, G and F mean greenhouse and field conditions respectively, the length of the red lines mean the influence of the different traits under greenhouse or field conditions. The influence for the traits at the specific stage is great; the influence will cause the great differences between cultivars. If cultivars are close to one line, it means these cultivars have advantages at this stage under this condition which the line shows.

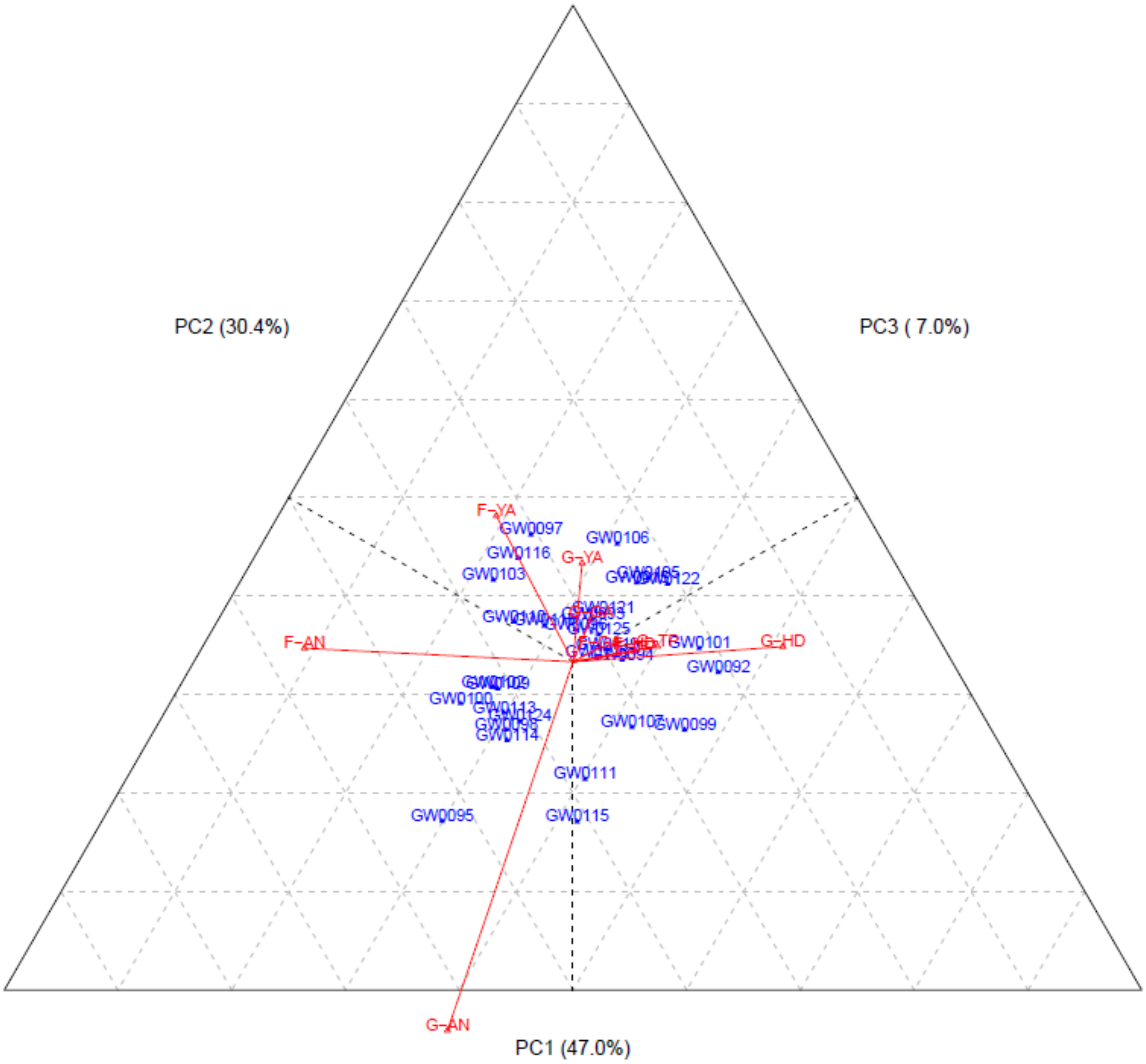

Figure S11. Three ways PCA analysis of F1 ovary size. In PCA analysis, G and F mean greenhouse and field conditions respectively, the length of the red lines mean the influence of the different traits under greenhouse or field conditions. The influence for the traits at the specific stage is great; the influence will cause the great differences between cultivars. If cultivars are close to one line, it means these cultivars have advantages at this stage under this condition which the line shows.

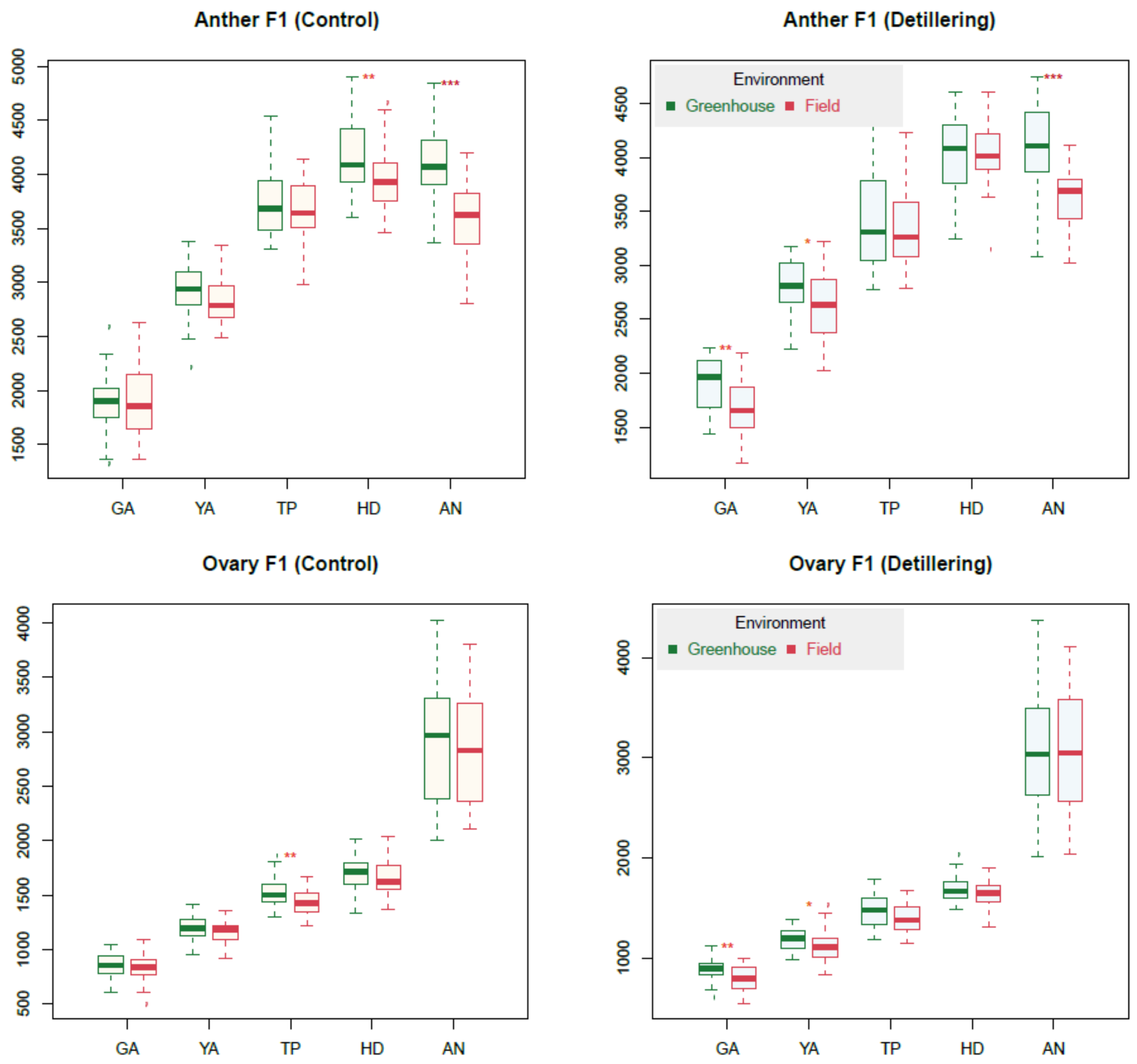

Figure S12. The influence of field and greenhouse on F1 anther and ovary size under control and detillering treatments. Significant difference is indicated by stars (**: p-value < 0.01; ***: p-value < 0.001).

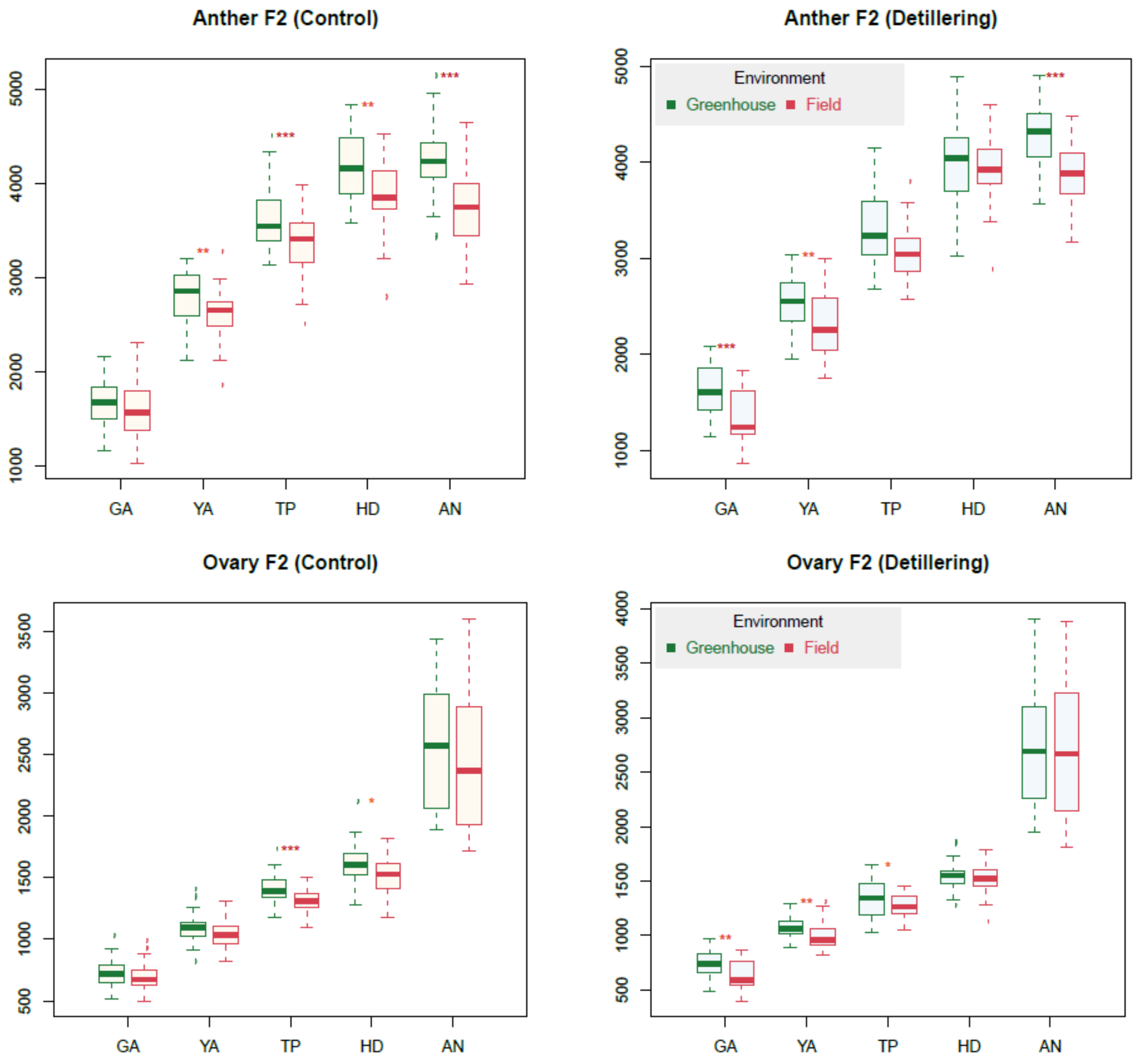

Figure S13. The influence of field and greenhouse on F2 anther and ovary size under control and detillering treatments. Significant difference is indicated by stars (**: p-value < 0.01; ***: p-value < 0.001).

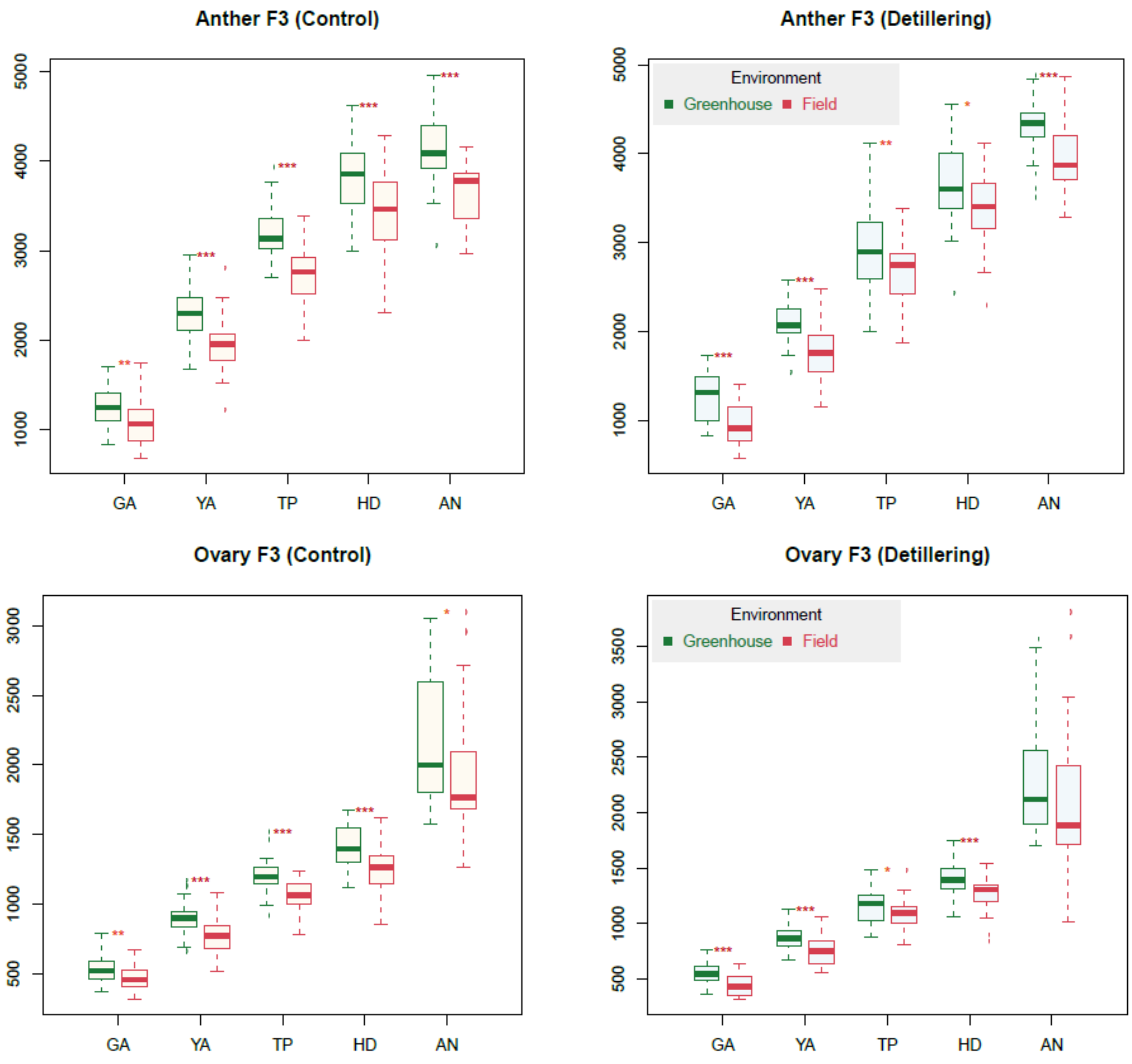

Figure S14. The influence of field and greenhouse on F3 anther and ovary size under control and detillering treatments. Significant difference is indicated by stars (**: p-value < 0.01; ***: p-value < 0.001).

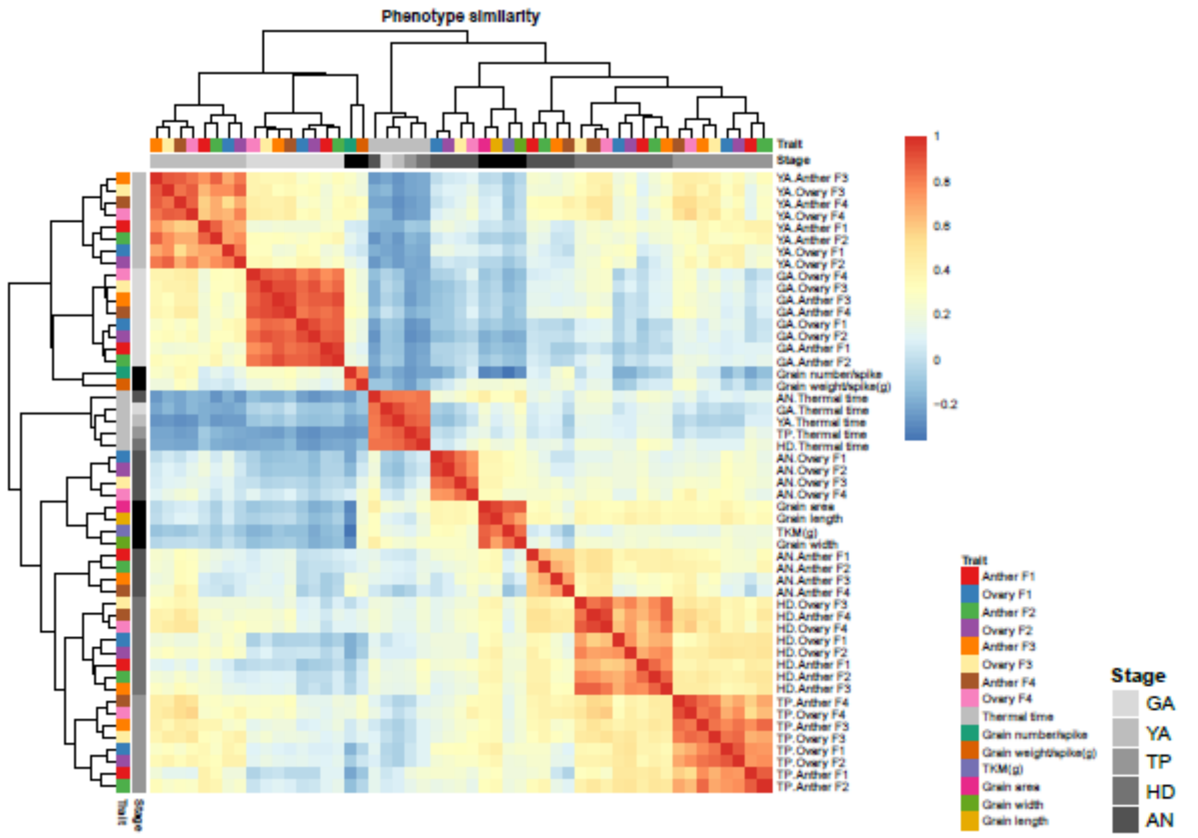

Figure S15. Phenotype similarity based on the anther size, ovary size, grain size and number. The distances between the traits reveal the connections oft he traits.

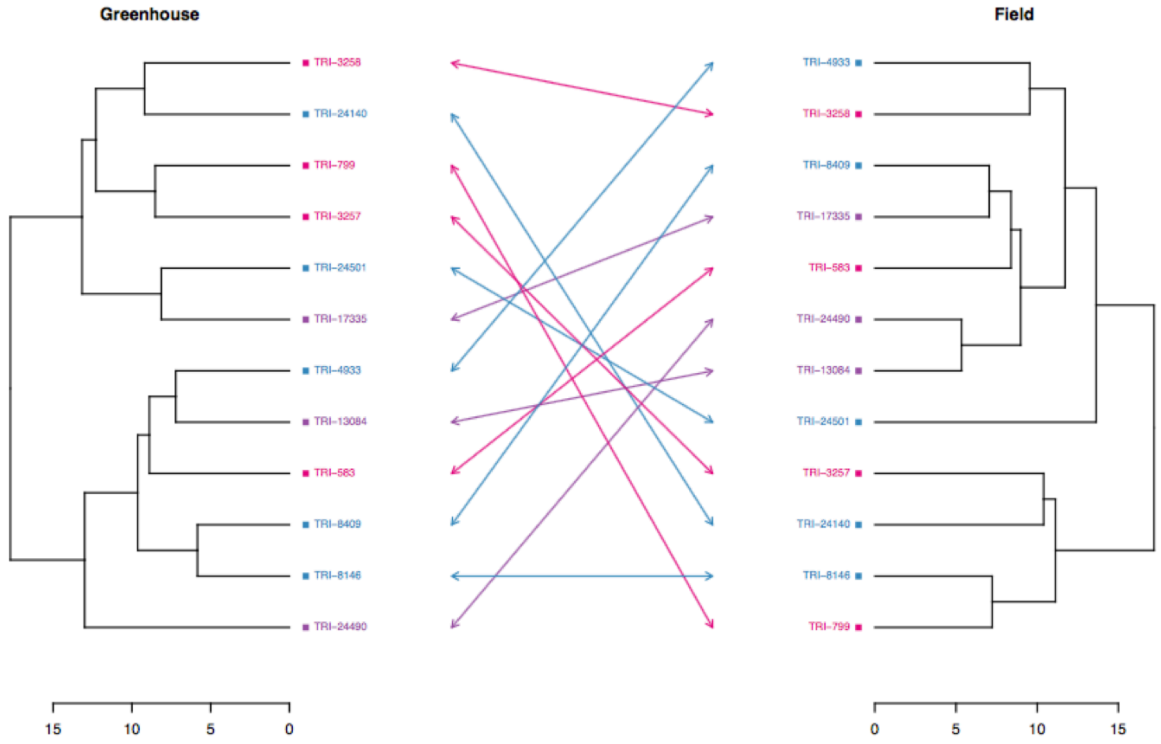

Figure S16. Phenotypic similarity trees showing the phenotypic relationship of genotypes in group 1, 2, 3 in field and greenhouse. The trees were constructed based on overall phenotypic distance matrices.

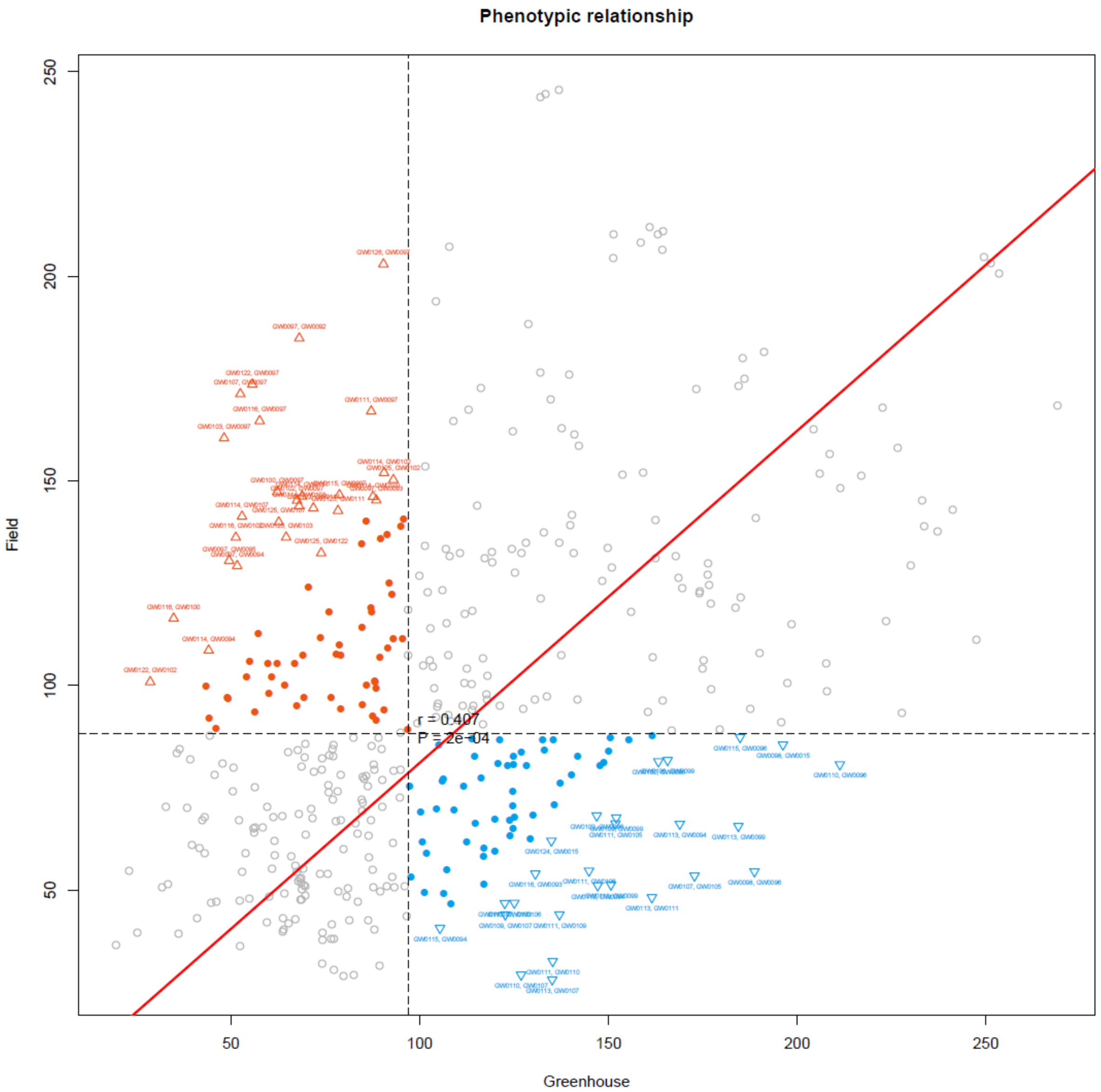

Figure S17. Scatter plot indicating the degree of correlation of phenotypic distance between genotypes under both field (x axis) and greenhouse conditions (y axis). Mantel test was performed to examine whether the phenotypic distances in the two conditions correlate with each other. P value was calculated with Monte-Carlo simulation (with 10,000 permutations). Genotype pairs that are far away from the regressed line (red) are labeled and colored (orange, small distances in control and large distances in stress; blue, otherwise).

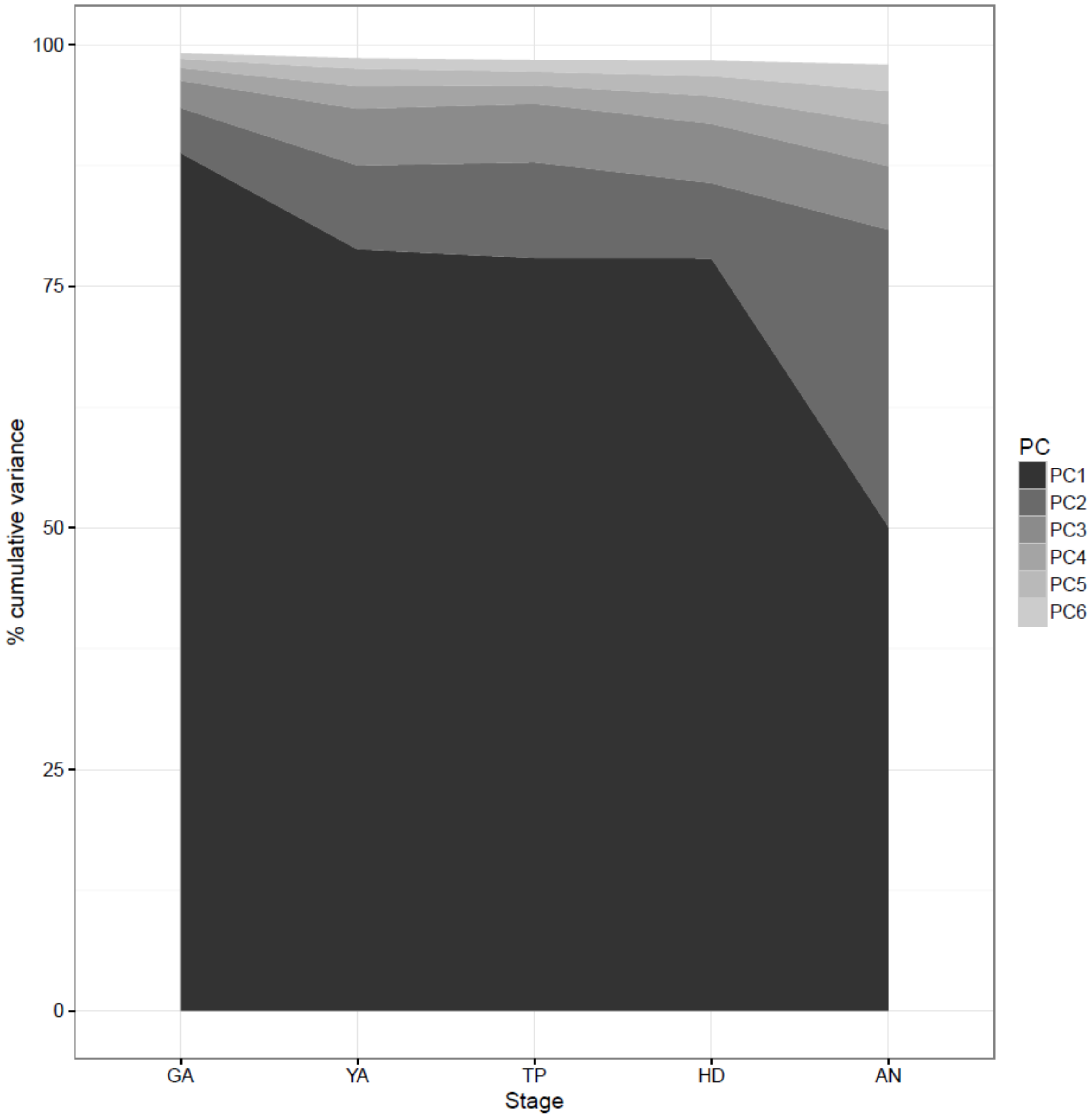

Figure S18. Projections of top six PCs based on PCA of phenotypic variance according the floret growth stages. The percentage of total explained variance is shown.

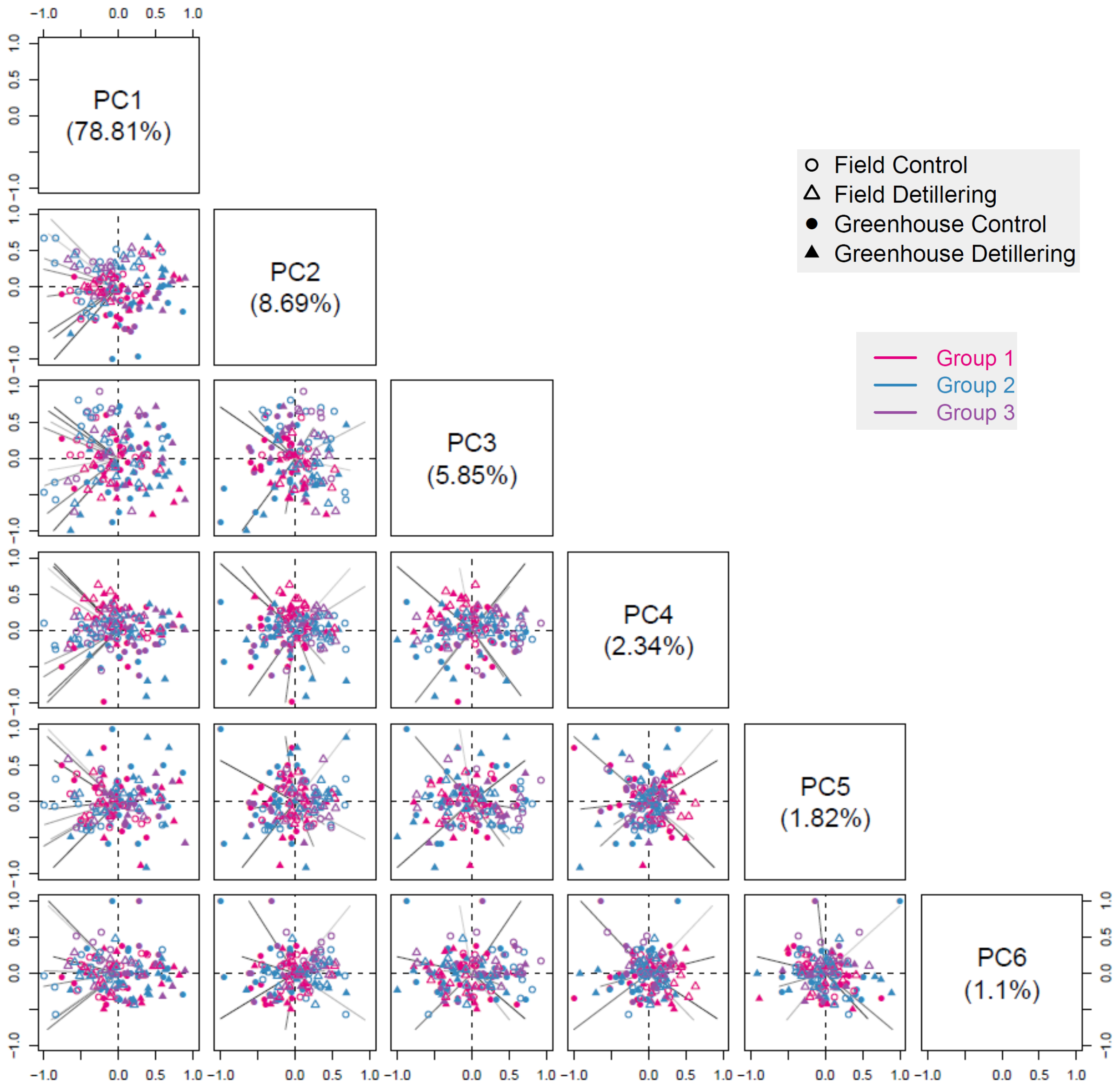

Figure S19. Scatter plots showing the principal component analysis (PCA) results at yellow anther (YA) stage (the phenotypic variance explained by the top six PCs).

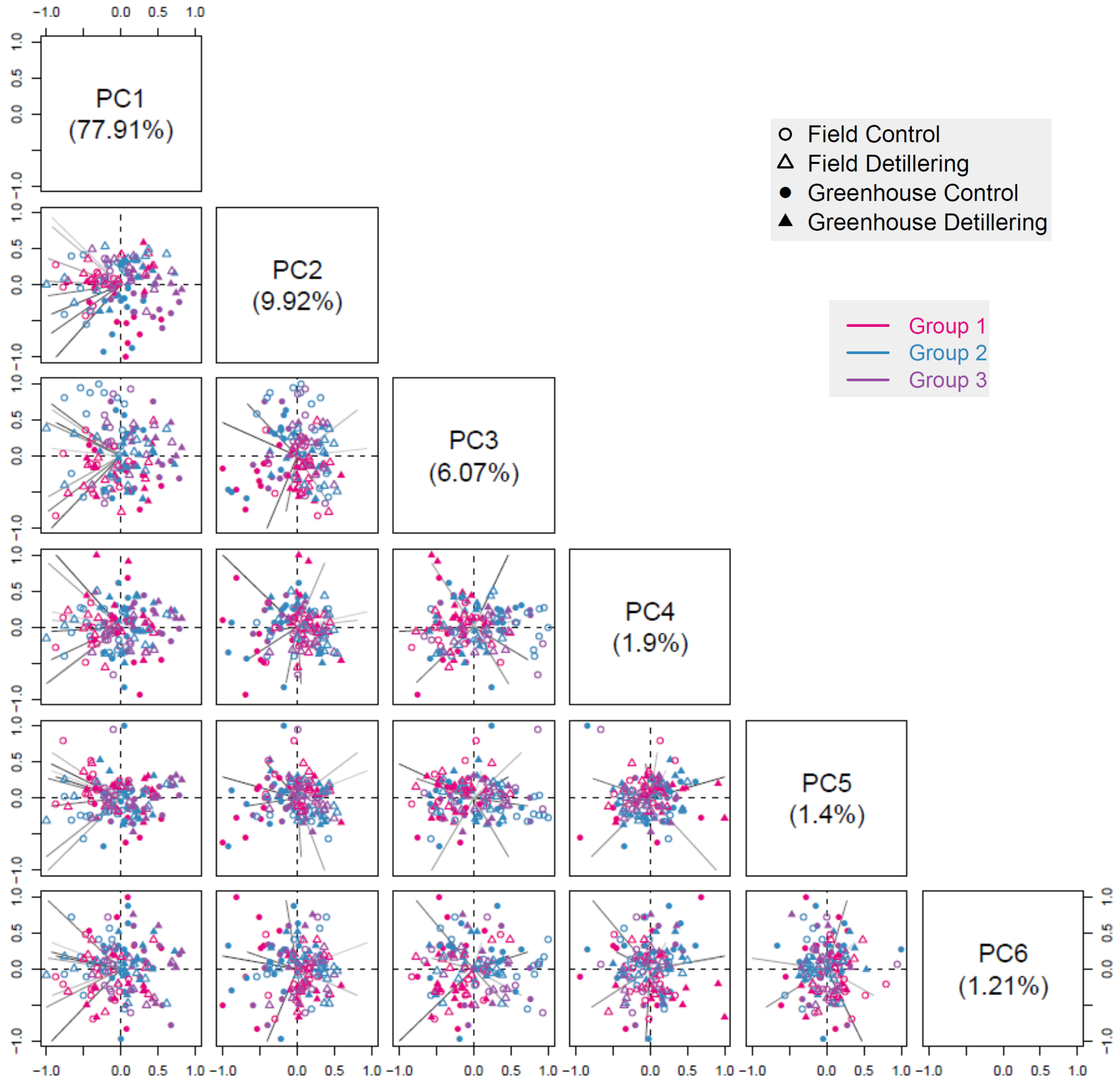

Figure S20. Scatter plots showing the principal component analysis (PCA) results at tipping (TP) stage (the phenotypic variance explained by the top six PCs).

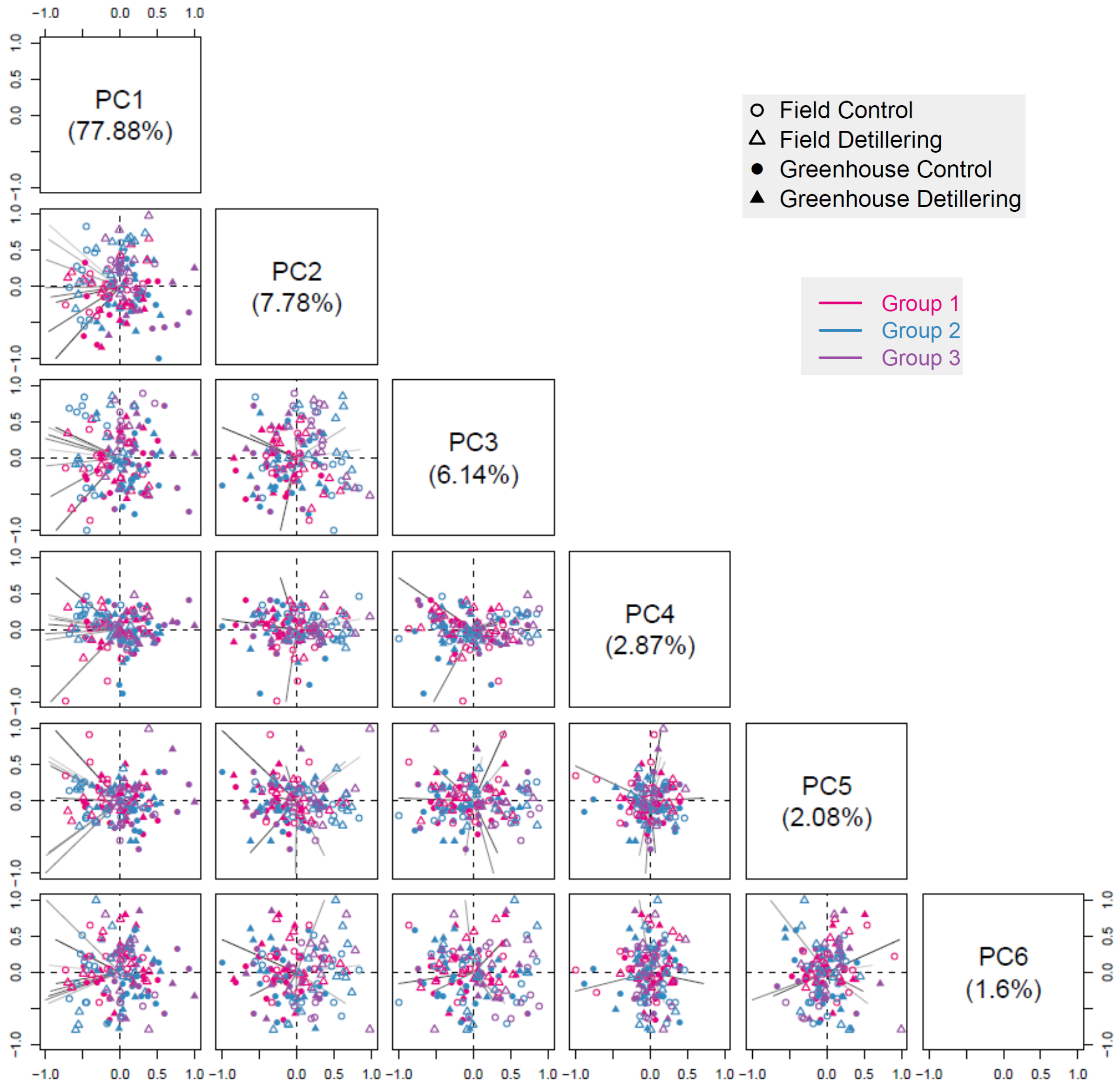

Figure S21. Scatter plots showing the principal component analysis (PCA) results at heading (HD) stage (the phenotypic variance explained by the top six PCs).

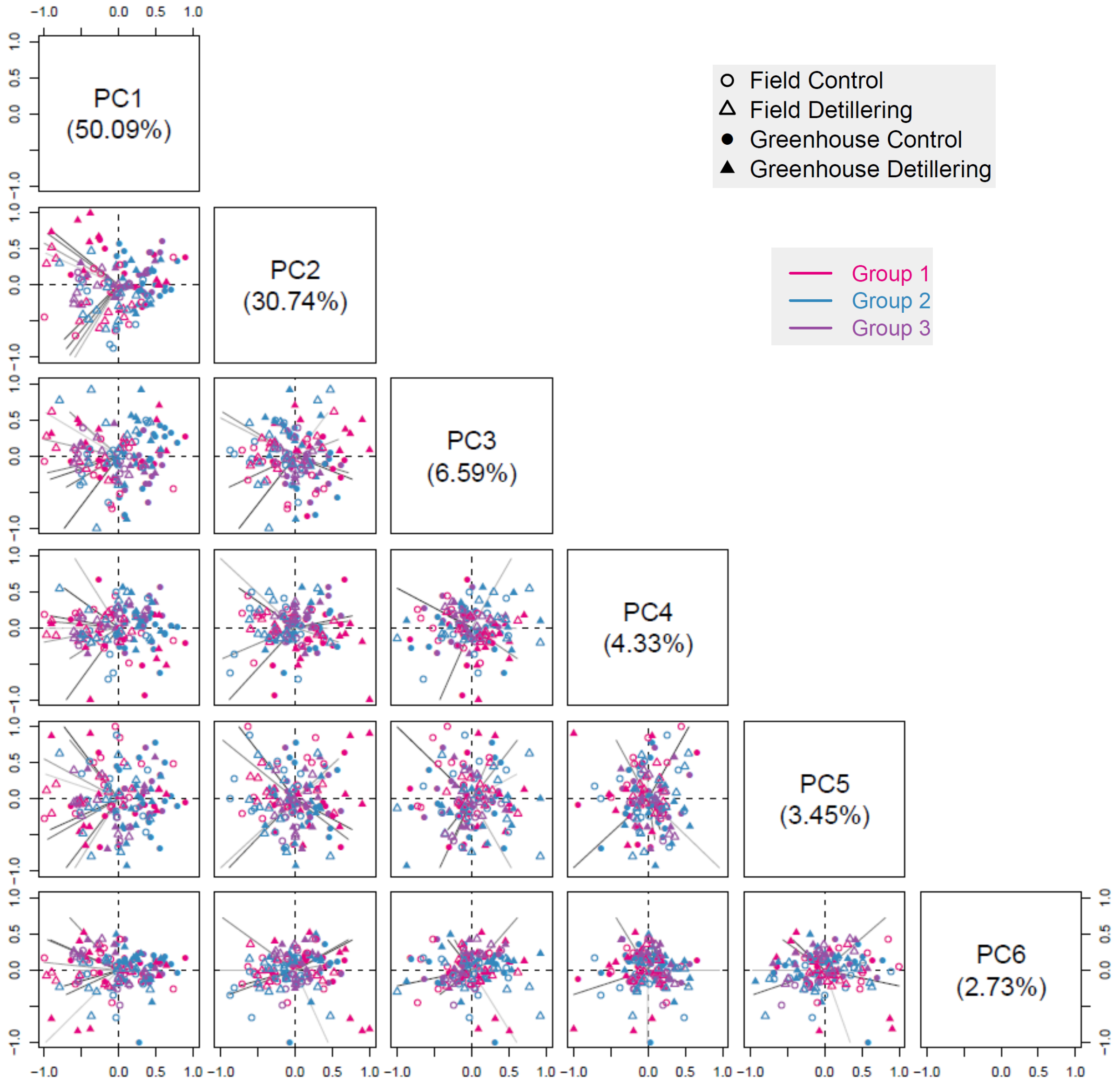

Figure S22. Scatter plots showing the principal component analysis (PCA) results at anthesis (AN) (the phenotypic variance explained by the top six PCs).
